# Bidirectional Mendelian Randomization Analysis Reveals Causal Associations Between Autoimmune Diseases and Colorectal Cancer

**DOI:** 10.14740/wjon2732

**Published:** 2026-03-05

**Authors:** Shuang Liu, Kun Chen, Yue Qi Wang, Xiao Yu Gu, Zhi He, Chen Zhang, Guo Qiu Wu, Su Su Luo, Xing Jin

**Affiliations:** a Center of Clinical Laboratory Medicine, Zhongda Hospital, Southeast University, Nanjing, Jiangsu, China; b Department of Laboratory Medicine, Medical School of Southeast University, Nanjing, Jiangsu, China; c The Third Department of Hepatic Surgery, Shanghai Eastern Hepatobiliary Surgery Hospital, Shanghai, China; d Moores Cancer Center, School of Medicine, University of California San Diego, La Jolla, CA, USA; e Department of Laboratory Medicine, The Affiliated Hospital of Yangzhou University, Yangzhou University, Yangzhou, China; f These authors contributed equally to this study.

**Keywords:** Colorectal cancer, Autoimmune diseases, Bidirectional Mendelian randomization, Genome-wide association study, Genetic causality, Celiac disease, Rheumatoid arthritis

## Abstract

**Background:**

Observational studies have reported associations between autoimmune diseases (AIDs) and colorectal cancer (CRC), but whether these relationships are causal remains unclear.

**Methods:**

We performed a bidirectional two-sample Mendelian randomization (MR) analysis to evaluate the causal effects of eight prevalent AIDs—systemic lupus erythematosus (SLE), rheumatoid arthritis (RA), ankylosing spondylitis (AS), gout, multiple sclerosis (MS), celiac disease (CD), eczema, and asthma—on CRC risk, and to examine the possibility of reverse causation. Genome-wide association study (GWAS) summary statistics from individuals of European ancestry were analyzed. The inverse-variance weighted (IVW) approach served as the primary MR estimator, with MR-Egger regression and the weighted median method applied as complementary analyses. Robustness was further evaluated through sensitivity analyses, including assessments of heterogeneity and horizontal pleiotropy.

**Results:**

Genetically predicted CD was associated with a reduced risk of CRC (IVW odds ratio (OR) = 0.94; 95% confidence interval (CI), 0.89–0.99; P = 0.028). Genetically predicted RA was associated with an increased risk of CRC (IVW OR = 1.06; 95% CI, 1.02–1.11; P = 0.004). No significant causal associations were observed for the other AIDs. Reverse MR provided no evidence that genetic liability to CRC causally influenced the risk of these AIDs. Sensitivity analyses supported the stability of the findings.

**Conclusions:**

This bidirectional MR study provides genetic evidence supporting unidirectional, modest causal effects of specific AIDs (CD and RA) on CRC risk. Further studies are warranted to clarify underlying mechanisms, including immune dysregulation, inflammation, and dietary factors, and to determine clinical implications.

## Introduction

Autoimmune diseases (AIDs) are characterized by dysregulated immune activation and chronic inflammation, processes that have long been implicated in carcinogenesis [1, 2]. In parallel, advances in cancer immunotherapy further underscored the biological overlap between autoimmunity and malignancy, as immune-related adverse events (irAEs) often resemble classical autoimmune phenotypes [3–7]. Together, these observations have intensified interest in the potential links between AIDs and cancer risk [1, 2, 7].

Colorectal cancer (CRC) represents a relevant disease model to explore this relationship, given the established contribution of intestinal inflammation to colorectal carcinogenesis [8, 9]. The intestinal immune system plays a central role in maintaining mucosal homeostasis and tumor surveillance, and immune dysregulation is frequently implicated in CRC-related inflammation-associated pathways [8–11]. Accordingly, previous population-based studies have evaluated whether chronic immune activation in AIDs may influence CRC risk, but the results remain inconsistent across diseases and study settings [8–10, 12]. For example, a large observational study involving 4.5 million male US veterans reported that patients with inflammatory bowel disease (IBD) had a significantly increased risk of developing CRC, while those with multiple sclerosis (MS) exhibited a reduced risk [9, 12]. In contrast, a cohort study using the French National Health Care Database found an elevated CRC risk among individuals with MS [11]. Thus, the association between AIDs and CRC remains unclear.

Furthermore, compelling evidence indicates that CRC itself can trigger rheumatic paraneoplastic syndromes, suggesting a bidirectional relationship between AIDs and CRC [13, 14]. Proposed mechanisms include tumor antigen-driven autoantibody generation and activation of sensitized lymphocytes, which may cross-react with host tissues, as well as tumor-derived factors that may trigger autoimmune-like manifestations [15]. Epidemiological findings from CRC screening settings further suggest potential links between CRC-related processes and subsequent autoimmune rheumatic diseases [16].

While observational studies have explored the relationship between AIDs and CRC, their ability to establish causality is constrained by confounding factors and reverse causation [17]. Mendelian randomization (MR), a robust genetic epidemiological method, overcomes these limitations by leveraging genetic variants as instrumental variables (IVs) for exposures [18–21]. Since genetic variants are randomly allocated at conception and fixed at birth, MR minimizes confounding and reduces the risk of reverse causality [18, 19, 21]. Bidirectional MR, an extension of this approach, further strengthens causal inference by evaluating relationships in both directions [20].

In this study, a bidirectional MR analysis was conducted to investigate the causal links between AIDs and CRC. Given the heterogeneity of AIDs in terms of pathogenesis and clinical features, we focused on eight common AIDs affecting diverse physiological systems: systemic lupus erythematosus (SLE), rheumatoid arthritis (RA), ankylosing spondylitis (AS), gout, MS, celiac disease (CD), eczema, and asthma. Using large-scale genome-wide association study (GWAS) summary data [22–30], we aimed to estimate the bidirectional causal relationships between these AIDs and CRC risk, assessing the effects of genetically predicted AIDs on CRC and the reverse effects of genetically predicted CRC on AIDs. We expected this analysis to provide genetic evidence that informs risk assessment and helps prioritize subsequent mechanistic and clinical investigations.

## Materials and Methods

### Study design

MR is based on three fundamental assumptions [21]. First, the relevance assumption, the IVs must be strongly associated with the exposure factor. Second, the independence assumption, the IVs must remain independent of any known or potential confounders affecting the exposure-outcome relationship. Third, the exclusion restriction assumption, which stipulates that IVs influence the outcome solely through the exposure factor and not via alternative biological pathways.

**Figure 1:**
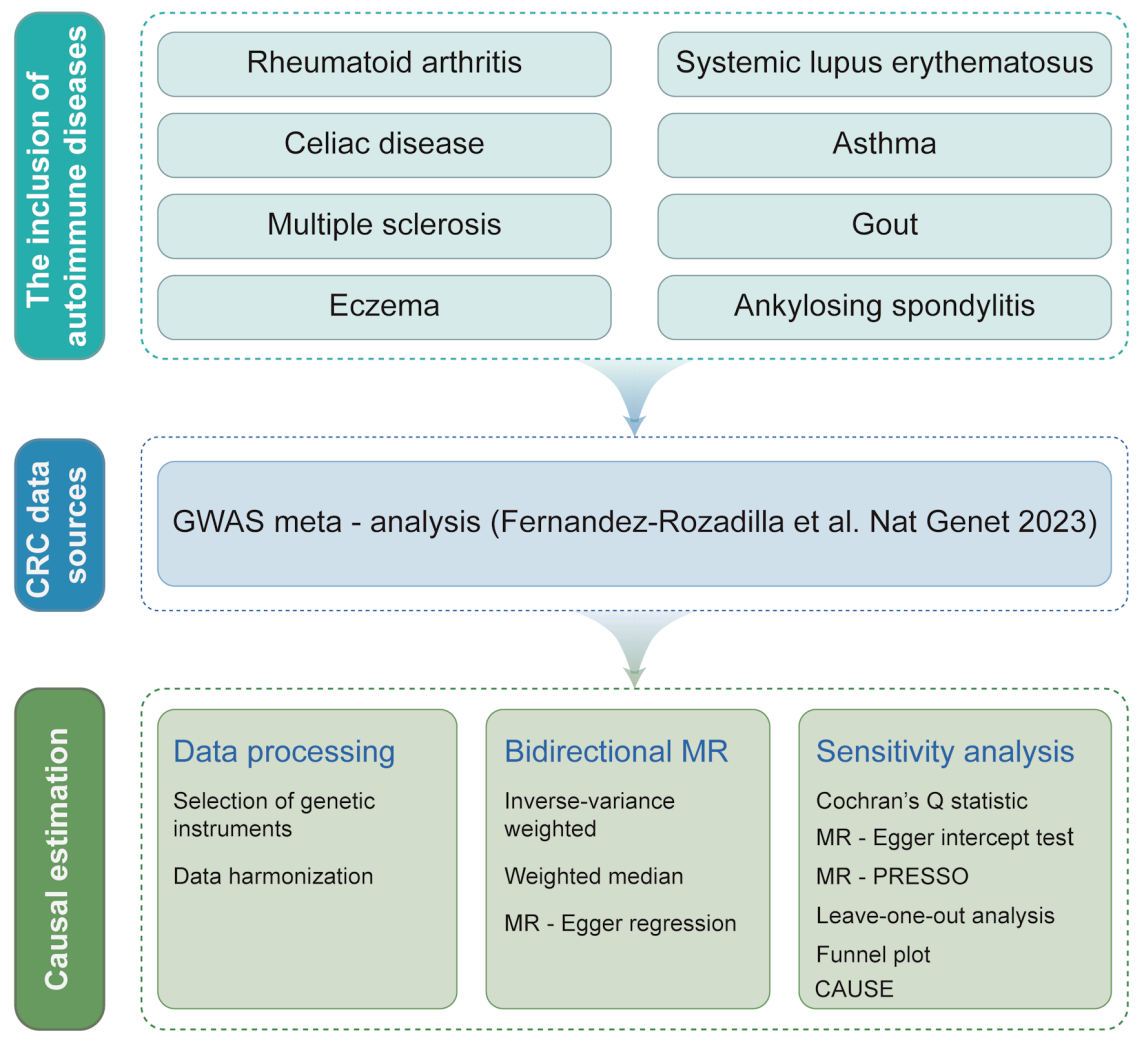
Overview of the bidirectional two-sample Mendelian randomization (MR) study design. Publicly available genome-wide association study (GWAS) summary statistics from European-ancestry populations were used for eight prevalent autoimmune diseases (AIDs)—rheumatoid arthritis, systemic lupus erythematosus, ankylosing spondylitis, gout, multiple sclerosis, celiac disease, eczema, and asthma—and for colorectal cancer (CRC), with CRC outcome data obtained from a GWAS meta-analysis (Fernandez-Rozadilla et al, 2023 [30]). For each AID (as the exposure) and in the reverse analysis with CRC as the exposure, we selected genetic instrumental variables (IVs) using standard MR criteria (genome-wide significant association with the exposure, linkage disequilibrium clumping to ensure independence, and harmonization of effect alleles across exposure and outcome datasets). Harmonized SNP–exposure and SNP–outcome associations were then analyzed in both directions to estimate causal effects. The inverse-variance weighted (IVW) method was used as the primary estimator, complemented by weighted median and MR-Egger regression to assess robustness under different assumptions. Multiple sensitivity analyses were performed to evaluate heterogeneity and potential horizontal pleiotropy, including Cochran’s Q statistic, MR-Egger intercept test, MR-pleiotropy residual sum and outlier (MR-PRESSO), leave-one-out analysis, funnel plot inspection, and Causal Analysis Using Summary Effect Estimates (CAUSE). All procedures and reporting followed the STROBE-MR guideline. SNP: single nucleotide polymorphism.

Figure 1 illustrates the study design [30]. Initially, publicly available GWAS data for eight prevalent AIDs and CRC were selected as data source. Subsequently, appropriate IVs that met established exposure criteria for both AIDs and CRC were selected for bidirectional MR analysis. The MR analysis was conducted using multiple methods to ensure the robustness of the results, followed by comprehensive sensitivity analyses to confirm the reliability of the findings. The entire study design and analyses process were conducted strictly by following the STROBE-MR guideline [31].

### Data sources for AIDs and CRC

Details of the GWAS datasets selected for this study are summarized in Table 1 [22–30]. All exposure and outcome data were derived from populations of European descent. Although the CRC GWAS included both European and East Asian participants, only individuals of European descent were retained for analysis to minimize population stratification bias. All studies included both male and female participants and provided large-scale genetic coverage, with most datasets comprising more than 5 million single-nucleotide polymorphisms (SNPs) and sample sizes exceeding 15,000 individuals.

**Table 1:** Characteristics of the GWAS on Autoimmune Diseases and Colorectal Cancer Used for MR Analyses

Variables	PubMed identification	Year	No. of cases	No. of control participants	Population	Sex	SNPs	Sample size
Rheumatoid arthritis [22]	23143596	2012	13,838	33,742	European	Males and females	112,654	47,580
Systemic lupus erythematosus [23]	26502338	2015	5,201	9,066	European	Males and females	7,071,163	14,267
Celiac disease [24]	20190752	2010	4,533	10,750	European	Males and females	518,292	15,283
Asthma [25]	29273806	2018	19,954	107,715	European	Males and females	1,999,262	127,669
Multiple sclerosis [26]	31604244	2019	47,429	68,374	European	Males and females	6,304,359	115,803
Gout [27]	23263486	2013	2,115	67,259	European	Males and females	5,057,528	69,374
Ankylosing spondylitis [28]	/	2021	1,462	164,682	European	Males and females	16,380,022	166,144
Eczema [29]	26482879	2015	10,788	30,047	European	Males and females	11,059,641	40,835
Colorectal cancer^a^ [30]	36539618	2023	78,473	107,143	European (73%) and Asian (27%)	Males and females	11,738,639	185,616

^a^The CRC data consist of Asian and European ancestry, from which European ancestry data were extracted for subsequent analysis to ensure uniformity of the sample. GWAS: genome-wide association study; MR: Mendelian randomization; SNPs: single-nucleotide polymorphisms.

GWAS summary statistics for AS were obtained from the FinnGen project, including 1,462 cases and 164,682 controls of European ancestry. GWAS data for all other AIDs were acquired from the corresponding GWAS meta-analysis [22–30].

### IVs selection

AIDs and CRC were treated as separate exposure traits, and genetic IVs were constructed independently for each. SNPs associated with each exposure at genome-wide significance (P < 5 × 10^–8^) were selected; when fewer than three variants were available, the threshold was relaxed to P < 5 × 10^–6^. Independence among IVs was ensured using linkage disequilibrium (LD) clumping using the PLINK algorithm, retaining SNPs with low LD (e.g., R^2^ < 0.001 or clump windows < 10,000 kb). SNPs with effect allele frequency (EAF) ≤ 0.01 were excluded, and instrument strength was assessed using the F statistic (F > 10).

To minimize potential confounding, SNPs associated with predefined risk factors were screened using PhenoScanner and excluded (for AIDs: smoking, alcohol intake, hyperlipidemia, hypertension, allergies, air pollution, and body mass index; for CRC: smoking, alcohol intake, hyperlipidemia, hypertension, and BMI). SNPs strongly associated with the outcome were additionally removed (P < 5 × 10^–5^). Exposure and outcome datasets were harmonized by aligning effect alleles, palindromic SNPs with ambiguous strand orientation were excluded or handled according to prespecified rules.

### MR analysis

Random effects inverse-variance weighted (IVW), MR-Egger, and weighted median (WM) were used for MR analysis [32]. IVW was used as the main analysis because it is the most robust analysis and can provide a modest estimate with the presence of heterogeneity. MR-Egger and WM were used to validate the robustness of IVW estimates. A significant estimate provided by IVW with the same direction of estimates provided by MR-Egger and WM was considered as a robust finding. In cases of inconsistencies between different MR analyses, a strict instrument P value threshold was used and recalculated.

### Sensitivity analysis

To identify heterogeneity and remove abnormal SNPs, several statistical methods were utilized, including Cochran’s Q statistic, funnel plots, and MR-pleiotropy residual sum and outlier (MR-PRESSO) method. Heterogeneity was specifically defined as a P < 0.05 in the Cochran Q test. Furthermore, MR-Egger intercept tests were conducted to adjust for horizontal pleiotropy. The Causal Analysis Using Summary Effect Estimates (CAUSE) method was employed to detect potential false positive errors arising from correlated horizontal pleiotropy [33]. By employing leave-one-out (LOO) analyses and repeating the IVW analysis, the influence of individual SNPs on the causality assessments could be evaluated.

### Statistical analysis

MR analyses were conducted in R software (version 4.2.1), primarily with the TwoSampleMR (version 0.5.6) and MR-PRESSO (version 1.0) package. Data visualization was performed using the ggplot2 R package. Statistical significance was determined by a P value < 0.05.

### Institutional Review Board (IRB) approval

IRB approval was not applicable. Ethical approval was not required for this study because it was based on publicly available, deidentified summary statistics. Each original GWAS had obtained relevant ethical approval and participant consent.

### Ethical compliance with human/animal study

This study was conducted using publicly available, de-identified summary-level GWAS data. The original studies were performed in accordance with the ethical standards of the responsible institutional committees and with the Helsinki Declaration (as applicable). No new human or animal experiments were conducted for this work.

## Results

### Selection of genetic instruments for AIDs and CRC

Following quality control and SNP filtering, 59 independent SNPs were identified as IVs for RA. Similarly, 44, 12, 18, 95, 7, 11, 12, and 64 SNPs were selected as IVs for SLE, CD, asthma, MS, gout, AS, eczema, and CRC, respectively. All SNPs exhibited F-statistics > 100, confirming their strength as IVs and minimizing potential weak instrument bias.

To assess potential pleiotropy, all candidate IVs were queried in the PhenoScanner database, and SNPs associated with predefined risk factors were excluded. Regarding AIDs, the query results indicated that all IVs for RA, asthma, and AS remain unaffected by risk factors. On the other hand, a total of 16 IVs were affected by risk factors in SLE (rs6679677, rs17849501, rs6671847, rs4916215, rs4274624, rs10048743, rs2431697, rs389884, rs58721818, rs35000415, rs2736332, rs7899626, rs7097397, rs597808, rs35251378, and rs3747093). Meanwhile, risk factors were noted to confound six IVs in MS (rs2546890, rs1112718, rs12147246, rs4796224, rs7207542, and rs4808760). Concerning CD, gout, and eczema, each condition exhibited one IV disrupted by the presence of risk factors, namely rs653178, rs1481012, and rs6062486, respectively. For CRC, three SNPs, namely rs11727676, rs653178, and rs11672900, were found to be confounded by risk factors. The final set of IVs were shown here (Supplementary Material 1, wjon.elmerpub.com).

### Causal effects of AIDs on CRC

**Figure 2:**
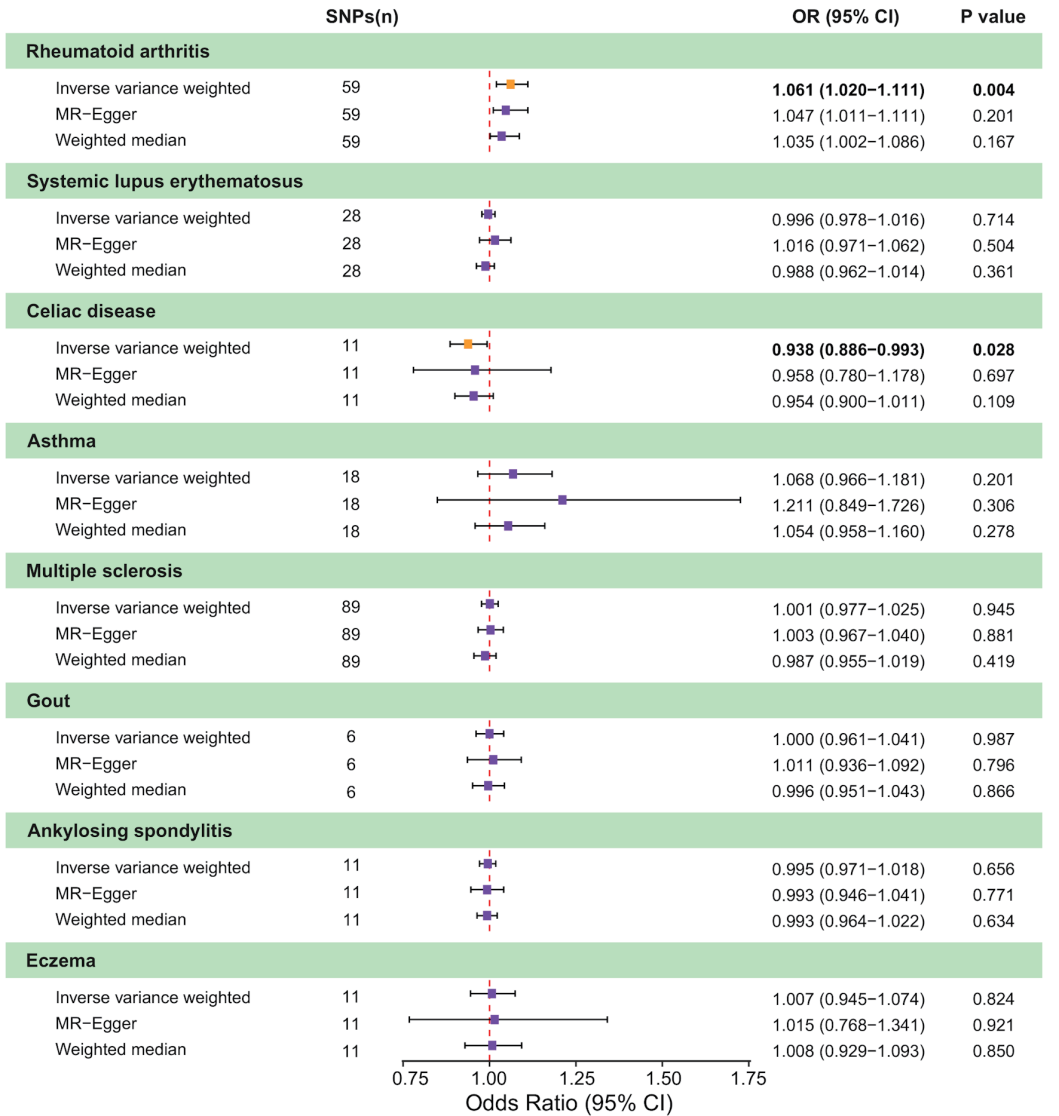
Causal effects of genetically predicted autoimmune diseases (AIDs) on colorectal cancer (CRC) risk in two-sample Mendelian randomization (MR) analyses. Forest plot shows odds ratio (OR) and 95% confidence interval (CI) for CRC per genetically predicted liability to eight AIDs: rheumatoid arthritis, systemic lupus erythematosus, celiac disease, asthma, multiple sclerosis, gout, ankylosing spondylitis, and eczema. For each AID, estimates are presented for the inverse-variance weighted (IVW) method, MR-Egger regression, and the weighted median approach, along with the corresponding number of instrumental SNPs and P values. Squares represent estimates and horizontal lines indicate 95% CIs; the vertical dashed line denotes the null effect (OR = 1.0). SNP: single nucleotide polymorphism.

To evaluate the influence of AIDs on CRC, we employed IVW as the primary method, complemented by MR Egger and WM. The MR estimates obtained from various methods are presented here (Fig. 2 and Supplementary Materials 2, 3, wjon.elmerpub.comm). The IVW analysis revealed a negative causal relationship between CD and CRC (odds ratio (OR) = 0.94; 95% confidence interval (CI), 0.89–0.99; P = 0.028). The results from the remaining MR methods indicated a consistent but nonsignificant trend (WM: OR = 0.95; 95% CI, 0.90–1.01; P = 0.109; MR-Egger: OR = 0.96; 95% CI, 0.78–1.18; P = 0.697). Additionally, a positive causal relationship was found between RA and CRC by IVW analysis (IVW: OR = 1.06; 95% CI, 1.02–1.11; P = 0.004). Other MR approaches showed consistent yet statistically nonsignificant patterns (WM: OR = 1.035; 95% CI, 1.002–1.086; P = 0.167; MR-Egger: OR = 1.047; 95% CI, 1.011–1.111; P = 0.201). No genetic causality was found between other diseases and CRC.

**Figure 3:**
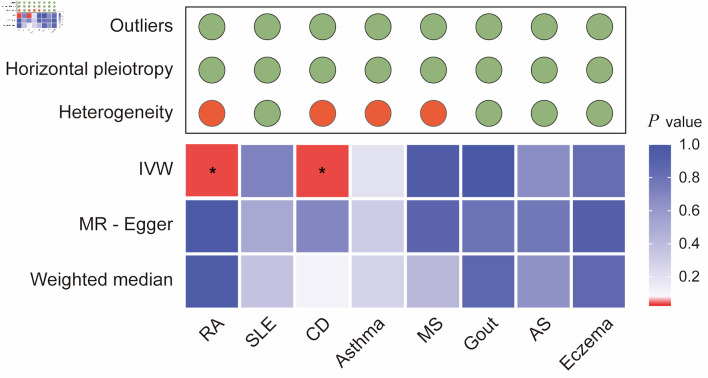
Integrated summary of Mendelian randomization (MR) estimates and sensitivity analyses for autoimmune diseases and colorectal cancer. The heatmap shows the strength of evidence from three MR methods (IVW, MR-Egger, and weighted median) for testing whether genetic susceptibility to eight autoimmune diseases (RA, SLE, CD, asthma, MS, gout, AS, and eczema) is causally related to colorectal cancer (CRC). The red boxes indicate the existence of a statistically significant causal relationship, whereas the blue boxes represent a lack of evidence for a causal relationship. The upper panel summarizes the results of sensitivity analyses, with green circles indicating no evidence and red circles indicating the presence of outliers, horizontal pleiotropy, or heterogeneity. *P value < 0.05. RA: rheumatoid arthritis; SLE: systemic lupus erythematosus; AS: ankylosing spondylitis; CD: celiac disease; IVW: inverse-variance weighted.

Sensitivity analyses were conducted to assess the robustness of the results. Heterogeneity tests (Fig. 3 and Supplementary Material 4, wjon.elmerpub.comm) indicated the presence of heterogeneity in CRC for four AIDs: RA (Cochran’s Q: IVW-P-_heterogeneity_ = 0.000, MR Egger-P-_heterogeneity_ = 0.000), CD (Cochran’s Q: IVW-P-_heterogeneity_ = 0.016, MR Egger-P-_heterogeneity_ = 0.010), asthma (Cochran’s Q: IVW-P-_heterogeneity_ = 0.000, MR Egger-P-_heterogeneity_ = 0.000), and MS (Cochran’s Q: IVW-P-_heterogeneity_ = 0.000, MR Egger-P-_heterogeneity_ = 0.000). Furthermore, the causal estimates showed no evidence of horizontal polymorphism (MR-Egger intercept P > 0.05) (Supplementary Material 4, wjon.elmerpub.comm).

Despite the observed heterogeneity, its magnitude was not sufficiently significant to invalidate MR estimates, as random effects IVW could counterbalance pooled heterogeneity. In addition, the LOO analysis failed to identify any outlier instruments, and the symmetry in the funnel plots supported the hypothesis of multiplicity of equilibria (Supplementary Materials 5, 6, wjon.elmerpub.comm). The CAUSE method was utilized in sensitivity analyses, revealing a significant causal association between prolonged genetically predicted CRC and CD (P = 0.021), as well as RA (P = 0.002).

### Causal effects of CRC on AIDs

**Figure 4:**
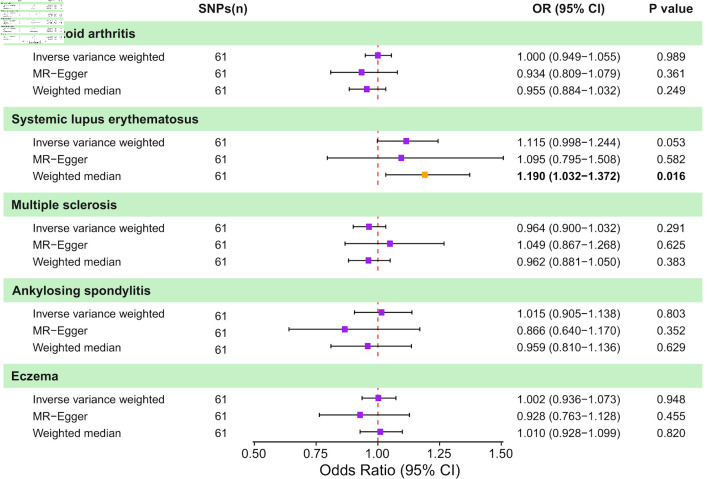
Causal effects of genetically predicted colorectal cancer (CRC) on autoimmune disease risk in two-sample Mendelian randomization (MR) analyses. Forest plot shows the odds ratio (OR) and 95% confidence interval (CI) for the genetically predicted liability of five autoimmune diseases to CRC. For each outcome, estimates are presented for the inverse-variance weighted (IVW) method, MR-Egger regression, and the weighted median approach, together with the corresponding number of instrumental SNPs and P values. Squares represent estimates and horizontal lines indicate 95% CIs; the vertical dashed line denotes the null effect (OR = 1.0). SNP: single nucleotide polymorphism.

**Figure 5:**
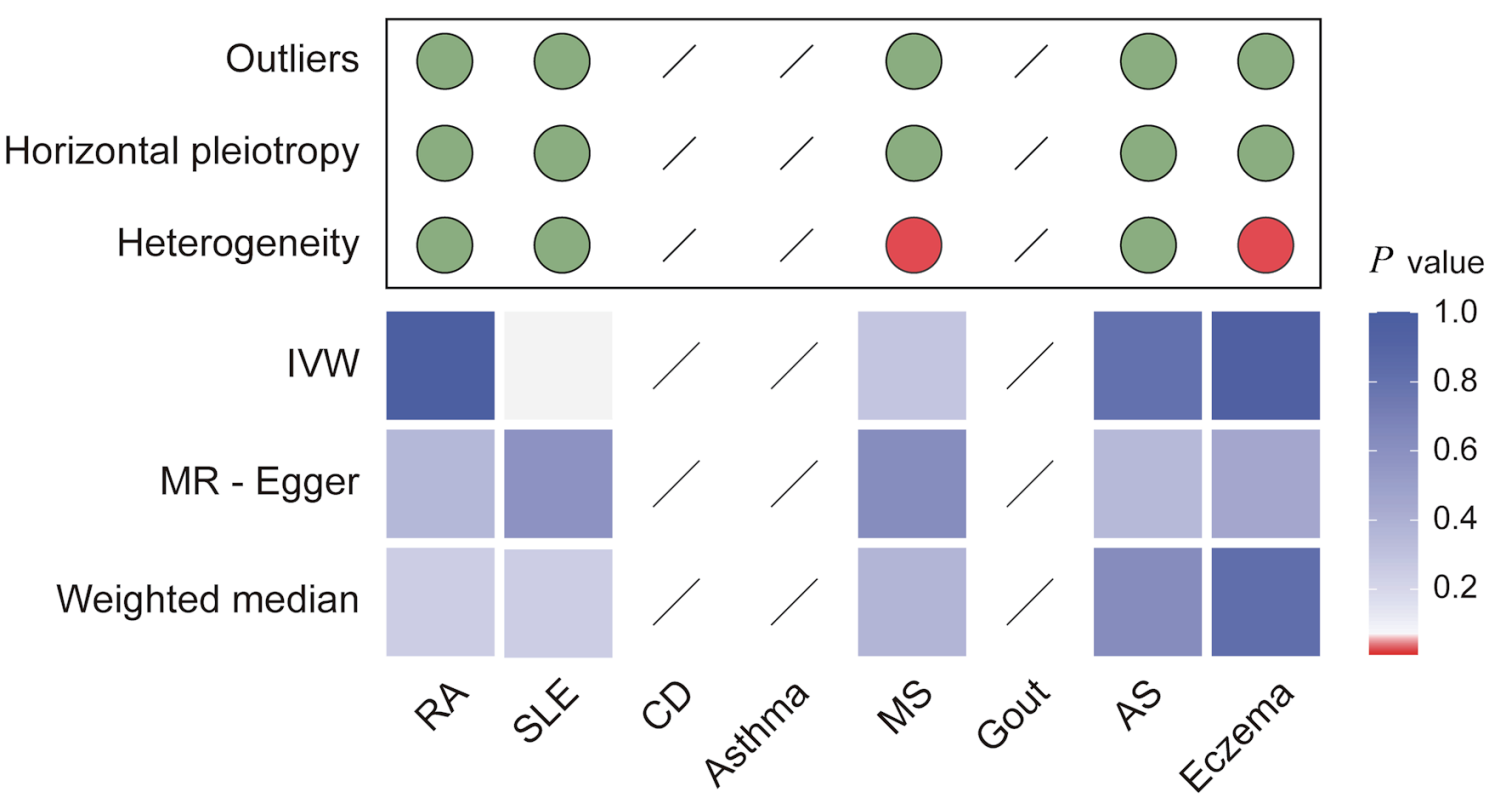
Integrated summary of Mendelian randomization (MR) estimates and sensitivity analyses for colorectal cancer and autoimmune diseases. The heatmap summarizes the strength of evidence from three MR methods (IVW, MR-Egger, and weighted median) for testing whether genetic liability to colorectal cancer (CRC) is causally associated with risk of eight autoimmune diseases (RA, SLE, CD, asthma, MS, gout, AS, and eczema). Red boxes indicate a statistically significant causal association (P < 0.05), whereas blue boxes represent a lack of evidence for a causal relationship. The upper panel summarizes sensitivity analyses, with green dots indicating no evidence and red dots indicating the presence of outliers, horizontal pleiotropy, or heterogeneity. Slashes denote an insufficient number of instrumental single-nucleotide polymorphisms (SNPs) to compute MR estimates for specific analyses. RA: rheumatoid arthritis; SLE: systemic lupus erythematosus; AS: ankylosing spondylitis; CD: celiac disease; IVW: inverse-variance weighted.

When CRC was used as exposure to test for bidirectional associations, no significant genetically predicted associations were identified (Fig. 4 and Supplementary Material 7, wjon.elmerpub.comm). However, a detailed examination revealed a trend towards a causal relationship between CRC and SLE (IVW: OR = 1.12; 95% CI, 1.00–1.24; P = 0.053; WM: OR = 1.19; 95% CI, 1.03–1.37; P = 0.016; MR-Egger: OR = 1.10; 95% CI, 0.80–1.51; P = 0.582). Sensitivity analyses revealed heterogeneity in MS (Cochran’s Q: IVW-P-_heterogeneity_ = 0.037, MR Egger-P-_heterogeneity_ = 0.037) and eczema (Cochran’s Q: IVW-P-_heterogeneity_ = 0.006, MR Egger-P-_heterogeneity_ = 0.006). A summary of the detailed MR and sensitivity analysis is displayed here (Fig. 5 and Supplementary Materials 8–11, wjon.elmerpub.comm).

## Discussion

In this study, we investigated the genetic causal relationships between multiple AIDs and CRC in a European population. Using MR analysis, we identified causal associations between CRC and CD and RA. Moreover, reverse MR analysis revealed that CRC does not serve as a risk factor for AIDs. Our findings introduced further evidence of genetic association between AIDs and CRC, providing new insights into the complex interplay between these conditions.

Our analysis suggested that generally predicted CD is associated with a reduced risk of CRC. CD is a chronic, multi-organ AID, primarily affecting the small intestine, typically triggered by the consumption of gluten-containing foods, and necessitating lifelong adherence to a gluten-free diet (GFD), which remains the only effective treatment strategy [34, 35]. According to previous studies, small bowel cancer (SBC) was more prevalent in patients with CD, but the link between CD and CRC remains controversial [36]. Two cohort studies conducted in Argentina and Sweden consistently demonstrated that patients with CD had an increased risk of CRC compared to the general population [37, 38]. Conversely, a study from Italy involving 1,757 patients found an association between CD and a decreased risk of CRC [39]. Moreover, a large number of studies have established that the risk of CRC in patients with CD aligns with that in the general population [40–42]. Our findings provide genetic evidence supporting a protective effect of CD on CRC risk, which may be partially explained by dietary and immunological factors associated with long-term adherence to a GFD. A GFD has been shown to alleviate symptoms and restore intestinal mucosal integrity in patients with CD [43]. A specific study involving 210 CD patients showed that those who adhered to a GFD for a minimum of 5 years did not exhibit an increased cancer risk, whereas those consuming a regular gluten-containing diet faced a higher risk for digestive tract cancers [44]. However, current evidence is insufficient to conclusively state that a GFD lowers the risk of CRC in the general population or among AIDs patients, suggesting the need for further high-quality clinical trials to determine its preventive effectiveness.

In contrast, our study identified a positive association between RA and CRC risk, which contrasts with findings from prior studies. For instance, Simon et al reported a reduced risk of CRC in patients with RA [45], as well as a regional cohort study from Taiwan found no overall increase in cancer risk [46]. These discrepancies may arise from several interrelated factors. On one hand, the cancer risk in RA patients is influenced by multiple confounding factors, including sample characteristics, duration and intensity of immunosuppressive therapy, and individual genetic susceptibility. On the other hand, chronic inflammation in RA stimulates persistent production of pro-tumorigenic mediators like interleukin-6 (IL-6), which may promote tumor migration and invasion despite potential protective effects from certain RA treatments [47].

For other AIDs, including MS and SLE, our MR analyses did not identify evidence of causal associations with CRC. Prior observational studies have reported conflicting results. In a retrospective study of matched cohorts, no differences were observed between individuals with and without MS in the incidence of CRC. In yet another retrospective study, MS patients were reported to have a significantly lower risk of CRC [48, 49]. In the case of SLE, a meta-analysis involving multiple types of cancer did not reveal a significant correlation between SLE and CRC [50]. Consequently, no evidence supports the association between other AIDs and CRC. Despite these findings, eliminating modifiable risk factors in patients with AIDs, especially those jointly associated with CRC, remains an important public health objective. It is, therefore, critical to conduct further research to determine the contribution of AIDs to the development of CRC.

Reverse MR analyses did not support a causal effect of CRC on the development of AIDs. Existing research in this area has largely focused on irAE associated with cancer immunotherapy and the exacerbation of pre-existing autoimmune conditions [51, 52]. In contrast, evidence regarding CRC itself as a causal trigger for autoimmune disease is limited. Our findings suggest that autoimmune-like manifestations reported in CRC patients are more likely to reflect paraneoplastic phenomena or treatment-related immune perturbations, rather than a direct causal pathway from CRC to AIDs.

In light of this and building on prior bidirectional MR work by Chen et al [53], our study provides a complementary and clinically motivated extension. First, the disease spectrum was expanded to include AS, gout, eczema, and asthma, which are common conditions considered by clinical experts to be relevant yet underexplored in relation to CRC risk. Second, our analyses and reporting were conducted in strict accordance with the STROBE-MR checklist, thereby improving transparency and facilitating comparability across studies. Notably, significant causal associations between genetic liability to CD and RA and CRC were observed, differing from prior bidirectional MR reports that found null effects; this discrepancy suggests that a single MR analysis may be insufficient to definitively confirm or exclude causal relationships. Instead, converging evidence from diverse GWAS datasets and independent MR methodologies is more effective in supporting clinical understanding and optimizing patient care. To further improve robustness to pleiotropy, the CAUSE framework was applied, which is designed to identify false-positive associations arising from correlated horizontal pleiotropy, thereby strengthening the credibility of the causal inferences.

Overall, our bidirectional MR findings should be viewed as hypothesis-generating genetic evidence rather than a basis for immediate changes in clinical practice, especially given the small but statistically robust genetic associations. A pragmatic next step is to combine genetic liability with key clinical modifiers—such as inflammatory activity, disease duration, medication exposure, and metabolic comorbidities—to build and validate CRC risk-stratification models for AIDs populations [54–57]. Importantly, these models should be tested in prospective, multi-ancestry cohorts to assess transportability, calibration, and real-world clinical utility across settings. Within this risk-prediction framework, future work can further examine whether adding mechanistically relevant signals improves prediction beyond standard clinical factors and whether these signals point to actionable translational opportunities. For example, repurposing drugs that act on immunometabolic pathways (e.g., glucagon-like peptide-1 (GLP-1)-based therapy and related incretin/ dipeptidyl peptidase-4 (DPP-4) signaling), or targeting circulating 20S proteasomes, may be worth evaluating as prevention-oriented strategies [58–66]. Notably, both approaches may exert immunomodulatory effects that could plausibly influence inflammation-linked carcinogenesis. However, any such extensions require independent replication and careful prospective validation before strong conclusions about cancer risk reduction can be drawn.

Several limitations of this study should be acknowledged. First, all GWAS data were derived from individuals of European ancestry, which may limit the generalizability of our findings to other ethnic groups or populations with different genetic backgrounds and lifestyle factors. Future studies using multi-ancestry datasets are warranted to validate these results. Furthermore, although MR strengthens causal inference by reducing confounding and reverse causation, it cannot fully disentangle mediation from horizontal pleiotropy. Some genetic variants may influence CRC risk through biological pathways independent of AIDs, potentially violating the exclusion restriction assumption despite extensive sensitivity analyses. Third, MR analyses are inherently limited in their ability to elucidate underlying biological mechanisms. While our findings suggest causal links between specific AIDs and CRC, they do not directly address mechanistic pathways such as alterations in intestinal immunity, chronic inflammation, or gut microbiota composition. Further experimental and prospective clinical studies are needed to clarify these mechanisms.

Despite these limitations, this study has several important strengths. By applying a bidirectional MR approach, we were able to investigate causal relationship between AIDs and CRC while minimizing bias from confounding and reverse causation that commonly affects observational studies. Moreover, this study includes a wide range of different AIDs, which enabled the exploration of the relationship between AIDs and CRC in a relatively comprehensive manner.

### Conclusions

In summary, this MR study provides genetic evidence that CD is causally associated with a reduced risk of CRC, whereas RA confers an increased risk. These findings enhance understanding of autoimmune and cancer interactions and may inform CRC risk assessment and prevention. Further mechanistic and prospective studies are needed to translate these genetic insights into clinical practice.

### Learning points

Genetically predicted CD is causally associated with a reduced risk of CRC. RA shows a positive genetic correlation with CRC. Bidirectional MR analysis provides robust evidence for unidirectional effects from autoimmune disease to CRC, without evidence of reverse causality.

## Supplementary Material

Suppl 1 SNPs used as genetic instruments for each studied disease.

Suppl 2 MR analyses of a causal association between genetic liability to autoimmune diseases and colorectal cancer.

Suppl 3 Scatter plots of the association between autoimmune diseases and colorectal cancer.

Suppl 4 Sensitivity analyses of a causal association between genetic liability to autoimmune diseases and colorectal cancer.

Suppl 5 Funnel plot analysis for the association between autoimmune diseases and colorectal cancer.

Suppl 6 Forest plots of leave-one-out analyses of the association between autoimmune diseases and colorectal cancer.

Suppl 7 MR analyses of a causal association between genetic liability to colorectal cancer and autoimmune diseases.

Suppl 8 Scatter plots of the association between colorectal cancer and autoimmune diseases.

Suppl 9 Funnel plot analysis for the association between colorectal cancer and autoimmune diseases.

Suppl 10 Forest plots of leave-one-out analyses of the association between colorectal cancer and autoimmune diseases.

Suppl 11 Sensitivity analyses of a causal association between genetic liability to colorectal cancer and autoimmune diseases.

## Data Availability

The authors declare that data supporting the findings of this study are available within the article.

## Abbreviations

IrAEs: immune-related adverse events
AIDs: autoimmune diseases
CRC: colorectal cancer
MR: Mendelian randomization
IBD: inflammatory bowel disease
MS: multiple sclerosis
RA: rheumatoid arthritis
SLE: systemic lupus erythematosus
AS: ankylosing spondylitis
CD: celiac disease
GWAS: genome-wide association studies
IV: instrumental variable
SNP: single nucleotide polymorphism
EAF: effect allele frequency
BMI: body mass index
WM: weighted median
IVW: inverse-variance weighted
CAUSE: Causal Analysis Using Summary Effect Estimates
LOO: leave-one-out
GFD: gluten-free diet
