# Supplementary material for: Bidirectional Mendelian Randomization Analysis Reveals Causal Associations Between Autoimmune Diseases and Colorectal Cancer
Source: World J Oncol. 2026 Mar 5;17(2):256–67. doi: 10.14740/wjon2732 (PMC12978415; doi:10.14740/wjon2732)
Supplement: Suppl 1 — SNPs used as genetic instruments for each studied disease. [file wjon-17-02-256-s001.docx]

| **Suppl 1. SNPs used as genetic instruments for each studied disease.** | | | | | | | | |  |
| --- | --- | --- | --- | --- | --- | --- | --- | --- | --- |
| **Disease** | **SNP** | **Effect allele** | **Other allele** | **EAF** | **Beta** | **SE** | ***P*** | **F-statistic^#^** |  |
| Ankylosing spondylitis | rs13033284 | C | T | 0.628 | -0.221 | 0.039 | 9.67E-09 | 3895.61 |  |
| Ankylosing spondylitis | rs9264277 | C | T | 0.730 | 0.520 | 0.045 | 3.73E-31 | 19820.69 |  |
| Ankylosing spondylitis | rs34982906 | C | T | 0.053 | 0.821 | 0.092 | 3.82E-19 | 12009.70 |  |
| Ankylosing spondylitis | rs16894011 | A | T | 0.073 | 2.108 | 0.089 | 5.32E-125 | 247789.80 |  |
| Ankylosing spondylitis | rs62394289 | A | G | 0.138 | 0.374 | 0.056 | 1.90E-11 | 5698.51 |  |
| Ankylosing spondylitis | rs79693223 | T | C | 0.045 | 1.295 | 0.106 | 3.36E-34 | 28023.80 |  |
| Ankylosing spondylitis | rs9378220 | A | C | 0.227 | -0.693 | 0.056 | 1.97E-35 | 33699.95 |  |
| Ankylosing spondylitis | rs112733823 | T | C | 0.217 | 0.360 | 0.047 | 1.43E-14 | 7640.80 |  |
| Ankylosing spondylitis | rs76644067 | A | G | 0.047 | 0.733 | 0.095 | 9.44E-15 | 8476.47 |  |
| Ankylosing spondylitis | rs181316459 | C | G | 0.047 | 0.985 | 0.100 | 1.02E-22 | 15874.19 |  |
| Ankylosing spondylitis | rs10807943 | C | T | 0.937 | -0.563 | 0.081 | 4.10E-12 | 6503.93 |  |
| Asthma | rs3771180 | T | G | 0.160 | -0.174 | 0.019 | 1.47E-20 | 1048.88 |  |
| Asthma | rs6893213 | T | C | 0.059 | 0.145 | 0.022 | 7.99E-11 | 301.21 |  |
| Asthma | rs7705042 | A | C | 0.333 | 0.080 | 0.013 | 8.53E-10 | 359.99 |  |
| Asthma | rs10455025 | C | A | 0.132 | 0.140 | 0.013 | 2.03E-25 | 578.41 |  |
| Asthma | rs20541 | G | A | 0.270 | -0.120 | 0.016 | 1.36E-14 | 724.45 |  |
| Asthma | rs3132600 | T | C | 0.082 | 0.116 | 0.020 | 1.33E-08 | 255.69 |  |
| Asthma | rs9272346 | A | G | 0.549 | 0.147 | 0.013 | 2.35E-28 | 1382.44 |  |
| Asthma | rs2325291 | A | G | 0.189 | -0.096 | 0.013 | 8.58E-13 | 357.55 |  |
| Asthma | rs10957979 | G | A | 0.465 | -0.074 | 0.013 | 2.33E-08 | 348.00 |  |
| Asthma | rs992969 | G | A | 0.216 | -0.158 | 0.014 | 4.27E-29 | 1091.06 |  |
| Asthma | rs1663687 | A | G | 0.267 | -0.084 | 0.013 | 1.52E-10 | 355.86 |  |
| Asthma | rs2155219 | T | G | 0.468 | 0.105 | 0.013 | 2.90E-15 | 704.81 |  |
| Asthma | rs167769 | T | C | 0.269 | 0.076 | 0.013 | 5.50E-09 | 290.27 |  |
| Asthma | rs17293632 | T | C | 0.099 | 0.118 | 0.015 | 8.81E-16 | 318.43 |  |
| Asthma | rs11071558 | G | A | 0.235 | -0.122 | 0.019 | 8.32E-11 | 686.81 |  |
| Asthma | rs12935657 | A | G | 0.143 | -0.104 | 0.015 | 2.06E-12 | 337.26 |  |
| Asthma | rs7209400 | T | C | 0.384 | 0.072 | 0.013 | 1.15E-08 | 315.42 |  |
| Asthma | rs2305479 | T | C | 0.311 | -0.178 | 0.013 | 1.00E-42 | 1762.08 |  |
| Celiac disease | rs2816316 | A | C | 0.202 | 0.254 | 0.036 | 1.45E-12 | 325.94 |  |
| Celiac disease | rs13003464 | G | A | 0.398 | 0.142 | 0.026 | 4.92E-08 | 148.01 |  |
| Celiac disease | rs1018326 | C | T | 0.421 | 0.152 | 0.026 | 3.78E-09 | 173.77 |  |
| Celiac disease | rs917997 | C | T | 0.306 | -0.232 | 0.030 | 5.97E-15 | 357.41 |  |
| Celiac disease | rs17810546 | G | A | 0.040 | 0.324 | 0.037 | 4.56E-18 | 123.06 |  |
| Celiac disease | rs13098911 | T | C | 0.093 | 0.278 | 0.042 | 2.53E-11 | 202.54 |  |
| Celiac disease | rs1464510 | A | C | 0.443 | 0.258 | 0.026 | 9.49E-24 | 517.86 |  |
| Celiac disease | rs13151961 | G | A | 0.066 | -0.324 | 0.038 | 6.31E-18 | 200.53 |  |
| Celiac disease | rs1738074 | C | T | 0.466 | -0.142 | 0.026 | 3.14E-08 | 155.73 |  |
| Celiac disease | rs2327832 | G | A | 0.095 | 0.232 | 0.030 | 1.41E-14 | 143.24 |  |
| Celiac disease | rs11221335 | C | T | 0.245 | 0.218 | 0.033 | 4.16E-11 | 271.88 |  |
| Eczema | rs3120745 | G | A | 0.691 | -0.107 | 0.020 | 4.73E-08 | 199.23 |  |
| Eczema | rs12144049 | T | C | 0.678 | -0.202 | 0.019 | 2.80E-27 | 734.15 |  |
| Eczema | rs6419573 | C | T | 0.709 | -0.124 | 0.020 | 2.92E-10 | 258.81 |  |
| Eczema | rs12188917 | C | T | 0.205 | 0.170 | 0.022 | 2.89E-15 | 385.74 |  |
| Eczema | rs4151657 | C | T | 0.337 | 0.102 | 0.018 | 7.86E-09 | 189.34 |  |
| Eczema | rs12334935 | A | G | 0.474 | 0.093 | 0.017 | 4.18E-08 | 174.06 |  |
| Eczema | rs2212434 | T | C | 0.451 | 0.129 | 0.017 | 2.09E-14 | 337.41 |  |
| Eczema | rs479844 | G | A | 0.548 | 0.144 | 0.017 | 3.45E-17 | 419.09 |  |
| Eczema | rs10790275 | C | G | 0.751 | 0.122 | 0.022 | 2.16E-08 | 230.08 |  |
| Eczema | rs8066625 | A | G | 0.107 | 0.176 | 0.032 | 3.84E-08 | 240.61 |  |
| Eczema | rs2918299 | T | C | 0.166 | 0.143 | 0.023 | 5.45E-10 | 217.76 |  |
| Gout | rs9311138 | G | A | 0.094 | -0.230 | 0.047 | 1.01E-06 | 633.18 |  |
| Gout | rs4475146 | A | C | 0.264 | -0.470 | 0.044 | 4.11E-26 | 6521.27 |  |
| Gout | rs17074636 | G | T | 0.044 | 0.710 | 0.140 | 6.82E-07 | 3095.24 |  |
| Gout | rs13287980 | A | G | 0.291 | 0.240 | 0.047 | 9.71E-07 | 1688.62 |  |
| Gout | rs8085724 | G | A | 0.988 | 1.800 | 0.360 | 1.04E-06 | 5721.72 |  |
| Gout | rs8087353 | T | C | 0.823 | -0.200 | 0.043 | 4.99E-06 | 817.60 |  |
| Multiple sclerosis | rs10801908 | T | C | 0.477 | -0.215 | 0.026 | 3.54E-16 | 2732.43 |  |
| Multiple sclerosis | rs59655222 | C | T | 0.137 | -0.123 | 0.019 | 3.76E-11 | 416.54 |  |
| Multiple sclerosis | rs478093 | G | A | 0.210 | 0.105 | 0.018 | 4.30E-09 | 426.44 |  |
| Multiple sclerosis | rs3737798 | G | A | 0.407 | -0.087 | 0.017 | 1.40E-07 | 426.50 |  |
| Multiple sclerosis | rs74449127 | G | A | 0.735 | -0.197 | 0.026 | 1.36E-14 | 1778.64 |  |
| Multiple sclerosis | rs6670198 | C | T | 0.441 | -0.145 | 0.018 | 2.03E-16 | 1213.24 |  |
| Multiple sclerosis | rs2317231 | T | G | 0.488 | -0.101 | 0.017 | 1.90E-09 | 588.23 |  |
| Multiple sclerosis | rs35486093 | G | A | 0.143 | 0.179 | 0.028 | 1.60E-10 | 922.73 |  |
| Multiple sclerosis | rs11809700 | T | C | 0.265 | 0.144 | 0.018 | 3.51E-15 | 948.95 |  |
| Multiple sclerosis | rs62195662 | C | T | 0.046 | 0.488 | 0.095 | 2.47E-07 | 2453.93 |  |
| Multiple sclerosis | rs35540610 | C | T | 0.217 | 0.135 | 0.019 | 2.88E-12 | 722.47 |  |
| Multiple sclerosis | rs1177228 | G | A | 0.323 | 0.107 | 0.019 | 8.57E-09 | 587.43 |  |
| Multiple sclerosis | rs7592560 | A | G | 0.567 | 0.104 | 0.017 | 2.87E-10 | 619.50 |  |
| Multiple sclerosis | rs12478539 | C | G | 0.213 | -0.123 | 0.019 | 4.37E-11 | 592.00 |  |
| Multiple sclerosis | rs12612620 | A | G | 0.225 | 0.212 | 0.036 | 4.26E-09 | 1839.27 |  |
| Multiple sclerosis | rs1014486 | C | T | 0.322 | 0.105 | 0.016 | 1.36E-10 | 560.54 |  |
| Multiple sclerosis | rs6763437 | A | G | 0.042 | -0.779 | 0.132 | 3.72E-09 | 5904.88 |  |
| Multiple sclerosis | rs11711621 | T | C | 0.758 | -0.104 | 0.019 | 5.63E-08 | 464.40 |  |
| Multiple sclerosis | rs2681424 | C | T | 0.484 | -0.121 | 0.017 | 2.71E-13 | 855.34 |  |
| Multiple sclerosis | rs13327021 | T | C | 0.445 | 0.115 | 0.017 | 1.73E-11 | 763.96 |  |
| Multiple sclerosis | rs9878602 | G | T | 0.308 | -0.084 | 0.016 | 2.60E-07 | 345.88 |  |
| Multiple sclerosis | rs4325907 | T | C | 0.327 | -0.099 | 0.017 | 3.68E-09 | 504.04 |  |
| Multiple sclerosis | rs438613 | C | T | 0.357 | 0.138 | 0.017 | 9.43E-17 | 1021.96 |  |
| Multiple sclerosis | rs6837324 | G | A | 0.225 | 0.086 | 0.017 | 3.16E-07 | 301.52 |  |
| Multiple sclerosis | rs9992763 | T | G | 0.380 | -0.090 | 0.016 | 4.51E-08 | 444.01 |  |
| Multiple sclerosis | rs2705616 | G | C | 0.366 | -0.083 | 0.016 | 4.35E-07 | 368.11 |  |
| Multiple sclerosis | rs17051321 | T | C | 0.191 | 0.095 | 0.019 | 4.95E-07 | 321.55 |  |
| Multiple sclerosis | rs67111717 | G | A | 0.410 | 0.096 | 0.019 | 4.96E-07 | 514.23 |  |
| Multiple sclerosis | rs10063294 | A | G | 0.479 | -0.099 | 0.016 | 1.13E-09 | 569.79 |  |
| Multiple sclerosis | rs11749040 | A | G | 0.091 | 0.197 | 0.023 | 3.54E-17 | 745.31 |  |
| Multiple sclerosis | rs9327104 | G | A | 0.980 | 0.310 | 0.061 | 4.41E-07 | 436.68 |  |
| Multiple sclerosis | rs9393975 | A | T | 0.203 | -0.766 | 0.043 | 1.49E-70 | 27145.22 |  |
| Multiple sclerosis | rs743771 | A | C | 0.485 | -0.099 | 0.016 | 8.55E-10 | 574.32 |  |
| Multiple sclerosis | rs72928038 | A | G | 0.071 | 0.161 | 0.025 | 9.01E-11 | 397.49 |  |
| Multiple sclerosis | rs2857700 | C | T | 0.105 | -0.767 | 0.024 | 1.00E-200 | 14459.62 |  |
| Multiple sclerosis | rs9277647 | T | C | 0.817 | -0.214 | 0.021 | 5.47E-24 | 1604.24 |  |
| Multiple sclerosis | rs1738074 | C | T | 0.466 | 0.114 | 0.017 | 9.91E-12 | 750.33 |  |
| Multiple sclerosis | rs56272720 | G | A | 0.953 | 0.479 | 0.091 | 1.63E-07 | 2411.75 |  |
| Multiple sclerosis | rs4896153 | A | T | 0.301 | -0.138 | 0.019 | 1.65E-13 | 939.64 |  |
| Multiple sclerosis | rs4947255 | T | C | 0.962 | -0.552 | 0.056 | 2.54E-23 | 2627.01 |  |
| Multiple sclerosis | rs114872782 | T | C | 0.981 | -2.450 | 0.255 | 7.72E-22 | 33174.86 |  |
| Multiple sclerosis | rs16822584 | T | C | 0.976 | -0.293 | 0.056 | 1.60E-07 | 465.60 |  |
| Multiple sclerosis | rs62420820 | A | G | 0.159 | 0.137 | 0.019 | 2.50E-13 | 587.13 |  |
| Multiple sclerosis | rs58546351 | G | A | 0.966 | -1.894 | 0.053 | 1.00E-200 | 35457.10 |  |
| Multiple sclerosis | rs12211604 | A | G | 0.286 | -0.093 | 0.018 | 1.86E-07 | 411.32 |  |
| Multiple sclerosis | rs60600003 | G | T | 0.045 | 0.134 | 0.026 | 4.20E-07 | 179.15 |  |
| Multiple sclerosis | rs2242508 | G | A | 0.480 | -0.089 | 0.017 | 2.94E-07 | 457.95 |  |
| Multiple sclerosis | rs354033 | A | G | 0.203 | -0.108 | 0.019 | 1.21E-08 | 438.53 |  |
| Multiple sclerosis | rs55970742 | T | C | 0.321 | -0.100 | 0.018 | 2.05E-08 | 510.90 |  |
| Multiple sclerosis | rs7385730 | T | G | 0.806 | -0.123 | 0.024 | 3.99E-07 | 552.57 |  |
| Multiple sclerosis | rs6990534 | G | A | 0.361 | 0.107 | 0.018 | 3.60E-09 | 616.84 |  |
| Multiple sclerosis | rs28703878 | G | A | 0.427 | 0.134 | 0.021 | 4.51E-10 | 1020.75 |  |
| Multiple sclerosis | rs7855251 | C | T | 0.390 | -0.110 | 0.020 | 4.23E-08 | 672.13 |  |
| Multiple sclerosis | rs1250551 | T | G | 0.282 | 0.116 | 0.017 | 2.66E-11 | 631.56 |  |
| Multiple sclerosis | rs11256593 | T | C | 0.360 | 0.186 | 0.017 | 6.78E-27 | 1881.96 |  |
| Multiple sclerosis | rs61884005 | G | C | 0.157 | -0.134 | 0.025 | 8.02E-08 | 553.66 |  |
| Multiple sclerosis | rs56232455 | A | G | 0.443 | 0.158 | 0.028 | 1.78E-08 | 1452.24 |  |
| Multiple sclerosis | rs12365699 | A | G | 0.070 | -0.144 | 0.023 | 3.15E-10 | 311.13 |  |
| Multiple sclerosis | rs4939490 | G | C | 0.384 | 0.137 | 0.017 | 4.25E-15 | 1032.17 |  |
| Multiple sclerosis | rs1204649 | C | A | 0.564 | -0.088 | 0.017 | 1.24E-07 | 442.38 |  |
| Multiple sclerosis | rs701006 | G | A | 0.495 | 0.114 | 0.017 | 1.35E-11 | 755.51 |  |
| Multiple sclerosis | rs7975763 | T | C | 0.247 | 0.121 | 0.021 | 7.80E-09 | 634.88 |  |
| Multiple sclerosis | rs1860545 | A | G | 0.220 | 0.117 | 0.017 | 7.79E-12 | 542.60 |  |
| Multiple sclerosis | rs3783196 | A | C | 0.392 | -0.083 | 0.016 | 4.34E-07 | 384.50 |  |
| Multiple sclerosis | rs9591325 | C | T | 0.033 | -0.212 | 0.034 | 4.16E-10 | 333.78 |  |
| Multiple sclerosis | rs17124032 | A | G | 0.082 | -0.217 | 0.032 | 7.08E-12 | 820.69 |  |
| Multiple sclerosis | rs34695601 | C | T | 0.096 | -0.109 | 0.020 | 3.16E-08 | 241.98 |  |
| Multiple sclerosis | rs12434551 | T | A | 0.486 | -0.104 | 0.016 | 1.83E-10 | 628.07 |  |
| Multiple sclerosis | rs6496663 | C | A | 0.390 | 0.101 | 0.018 | 2.78E-08 | 560.35 |  |
| Multiple sclerosis | rs35703946 | A | G | 0.087 | -0.173 | 0.029 | 1.94E-09 | 552.80 |  |
| Multiple sclerosis | rs3809627 | A | C | 0.462 | -0.097 | 0.018 | 3.25E-08 | 543.65 |  |
| Multiple sclerosis | rs17724508 | C | T | 0.196 | -0.208 | 0.038 | 5.30E-08 | 1598.80 |  |
| Multiple sclerosis | rs7190580 | G | A | 0.265 | -0.098 | 0.018 | 4.64E-08 | 435.59 |  |
| Multiple sclerosis | rs6564681 | T | C | 0.450 | -0.094 | 0.018 | 1.80E-07 | 513.20 |  |
| Multiple sclerosis | rs415759 | C | T | 0.811 | 0.119 | 0.022 | 4.76E-08 | 502.86 |  |
| Multiple sclerosis | rs7200146 | T | G | 0.371 | -0.171 | 0.017 | 7.00E-24 | 1597.85 |  |
| Multiple sclerosis | rs12925972 | C | T | 0.451 | 0.095 | 0.017 | 3.07E-08 | 515.34 |  |
| Multiple sclerosis | rs1026916 | G | A | 0.462 | -0.130 | 0.017 | 1.02E-13 | 975.86 |  |
| Multiple sclerosis | rs2150879 | A | G | 0.375 | -0.104 | 0.016 | 3.29E-10 | 584.72 |  |
| Multiple sclerosis | rs9955954 | G | A | 0.256 | -0.110 | 0.019 | 1.54E-08 | 536.04 |  |
| Multiple sclerosis | rs1077667 | T | C | 0.188 | -0.152 | 0.021 | 8.37E-13 | 820.81 |  |
| Multiple sclerosis | rs11666263 | G | A | 0.691 | -0.103 | 0.018 | 1.20E-08 | 525.34 |  |
| Multiple sclerosis | rs1465697 | T | C | 0.327 | 0.124 | 0.019 | 3.48E-11 | 793.23 |  |
| Multiple sclerosis | rs6032662 | T | C | 0.228 | -0.134 | 0.018 | 2.85E-13 | 734.32 |  |
| Multiple sclerosis | rs6742 | C | T | 0.212 | 0.159 | 0.030 | 1.30E-07 | 988.61 |  |
| Multiple sclerosis | rs6012503 | G | A | 0.511 | 0.088 | 0.017 | 1.62E-07 | 447.23 |  |
| Multiple sclerosis | rs2248461 | A | G | 0.382 | -0.108 | 0.017 | 5.33E-10 | 642.77 |  |
| Multiple sclerosis | rs9610458 | T | C | 0.457 | 0.114 | 0.017 | 4.57E-12 | 754.77 |  |
| Multiple sclerosis | rs140522 | C | T | 0.381 | -0.111 | 0.018 | 2.85E-10 | 671.97 |  |
| Rheumatoid arthritis | rs2301888 | A | G | 0.363 | -0.128 | 0.017 | 2.20E-18 | 436.19 |  |
| Rheumatoid arthritis | rs6679677 | A | C | 0.026 | 0.593 | 0.022 | 2.10E-149 | 1030.36 |  |
| Rheumatoid arthritis | rs2105325 | C | A | 0.164 | 0.105 | 0.017 | 3.10E-10 | 175.15 |  |
| Rheumatoid arthritis | rs187786174 | A | G | 0.499 | -0.117 | 0.017 | 3.30E-14 | 391.61 |  |
| Rheumatoid arthritis | rs2235924 | A | G | 0.687 | -0.094 | 0.017 | 3.60E-09 | 219.99 |  |
| Rheumatoid arthritis | rs12126142 | A | G | 0.293 | -0.083 | 0.016 | 3.50E-09 | 165.37 |  |
| Rheumatoid arthritis | rs2317230 | T | G | 0.430 | 0.077 | 0.014 | 2.10E-08 | 166.77 |  |
| Rheumatoid arthritis | rs28411352 | T | C | 0.217 | 0.113 | 0.013 | 3.60E-12 | 250.85 |  |
| Rheumatoid arthritis | rs1858036 | G | A | 0.438 | -0.113 | 0.014 | 1.20E-14 | 364.55 |  |
| Rheumatoid arthritis | rs3087243 | A | G | 0.369 | -0.139 | 0.017 | 1.70E-22 | 522.05 |  |
| Rheumatoid arthritis | rs6712515 | C | T | 0.477 | -0.104 | 0.014 | 6.70E-15 | 312.96 |  |
| Rheumatoid arthritis | rs11889341 | T | C | 0.240 | 0.131 | 0.018 | 6.70E-19 | 361.02 |  |
| Rheumatoid arthritis | rs10175798 | A | G | 0.443 | 0.086 | 0.014 | 5.40E-09 | 210.66 |  |
| Rheumatoid arthritis | rs12466919 | T | C | 0.638 | 0.113 | 0.013 | 5.70E-13 | 341.77 |  |
| Rheumatoid arthritis | rs5019428 | A | G | 0.523 | 0.086 | 0.014 | 7.20E-10 | 213.05 |  |
| Rheumatoid arthritis | rs3806624 | G | A | 0.263 | 0.083 | 0.011 | 1.90E-08 | 154.65 |  |
| Rheumatoid arthritis | rs73081554 | T | C | 0.018 | 0.166 | 0.029 | 4.60E-08 | 56.04 |  |
| Rheumatoid arthritis | rs11933540 | C | T | 0.273 | 0.139 | 0.018 | 8.80E-17 | 443.98 |  |
| Rheumatoid arthritis | rs13142500 | C | T | 0.461 | 0.094 | 0.017 | 5.00E-09 | 254.30 |  |
| Rheumatoid arthritis | rs2561477 | A | G | 0.190 | -0.083 | 0.011 | 1.90E-09 | 122.70 |  |
| Rheumatoid arthritis | rs7731626 | A | G | 0.229 | -0.186 | 0.018 | 7.30E-24 | 710.99 |  |
| Rheumatoid arthritis | rs9348832 | A | G | 0.028 | 0.255 | 0.031 | 5.20E-19 | 201.48 |  |
| Rheumatoid arthritis | rs9277956 | C | G | 0.153 | 0.223 | 0.020 | 7.80E-32 | 749.33 |  |
| Rheumatoid arthritis | rs1611236 | A | G | 0.330 | -0.117 | 0.011 | 2.10E-15 | 346.01 |  |
| Rheumatoid arthritis | rs7752903 | G | T | 0.039 | 0.329 | 0.029 | 2.70E-26 | 461.81 |  |
| Rheumatoid arthritis | rs9258357 | C | T | 0.077 | 0.174 | 0.019 | 5.30E-23 | 248.83 |  |
| Rheumatoid arthritis | rs9277411 | T | C | 0.687 | -0.288 | 0.013 | 1.50E-85 | 2114.42 |  |
| Rheumatoid arthritis | rs9296009 | T | A | 0.201 | 0.528 | 0.018 | 1.00E-200 | 5632.09 |  |
| Rheumatoid arthritis | rs2858329 | G | A | 0.514 | 0.446 | 0.016 | 1.00E-200 | 6329.97 |  |
| Rheumatoid arthritis | rs2233424 | T | C | 0.067 | 0.231 | 0.028 | 7.60E-19 | 384.50 |  |
| Rheumatoid arthritis | rs212389 | A | G | 0.324 | 0.095 | 0.018 | 3.30E-10 | 228.66 |  |
| Rheumatoid arthritis | rs6930468 | G | A | 0.350 | 0.094 | 0.011 | 5.50E-11 | 232.73 |  |
| Rheumatoid arthritis | rs112733823 | T | C | 0.872 | 0.285 | 0.019 | 6.60E-39 | 1060.61 |  |
| Rheumatoid arthritis | rs7754520 | T | C | 0.090 | -0.462 | 0.024 | 9.00E-67 | 2064.81 |  |
| Rheumatoid arthritis | rs76153210 | T | C | 0.984 | 0.174 | 0.025 | 2.20E-09 | 54.29 |  |
| Rheumatoid arthritis | rs1571878 | T | C | 0.388 | -0.151 | 0.012 | 6.10E-30 | 625.58 |  |
| Rheumatoid arthritis | rs3778753 | G | A | 0.343 | 0.105 | 0.017 | 1.10E-14 | 288.16 |  |
| Rheumatoid arthritis | rs2736337 | C | T | 0.373 | 0.105 | 0.011 | 4.80E-12 | 298.98 |  |
| Rheumatoid arthritis | rs11574914 | A | G | 0.200 | 0.113 | 0.018 | 2.10E-13 | 236.48 |  |
| Rheumatoid arthritis | rs1953126 | C | T | 0.286 | -0.086 | 0.014 | 1.00E-09 | 174.38 |  |
| Rheumatoid arthritis | rs947474 | A | G | 0.186 | 0.104 | 0.018 | 1.50E-08 | 189.61 |  |
| Rheumatoid arthritis | rs706778 | T | C | 0.499 | 0.086 | 0.014 | 1.50E-10 | 213.50 |  |
| Rheumatoid arthritis | rs71508903 | T | C | 0.149 | 0.157 | 0.017 | 2.30E-20 | 359.53 |  |
| Rheumatoid arthritis | rs73013527 | T | C | 0.327 | -0.094 | 0.017 | 9.80E-11 | 225.17 |  |
| Rheumatoid arthritis | rs11217044 | C | T | 0.203 | -0.131 | 0.018 | 3.60E-15 | 319.76 |  |
| Rheumatoid arthritis | rs4409785 | C | T | 0.125 | 0.105 | 0.017 | 3.00E-08 | 139.44 |  |
| Rheumatoid arthritis | rs773125 | G | A | 0.341 | -0.086 | 0.014 | 4.40E-10 | 191.75 |  |
| Rheumatoid arthritis | rs9603616 | T | C | 0.266 | -0.105 | 0.017 | 4.60E-12 | 249.51 |  |
| Rheumatoid arthritis | rs3784099 | A | G | 0.397 | -0.094 | 0.017 | 7.10E-10 | 244.94 |  |
| Rheumatoid arthritis | rs168962 | G | C | 0.766 | -0.083 | 0.016 | 1.70E-08 | 142.96 |  |
| Rheumatoid arthritis | rs8026898 | A | G | 0.316 | 0.148 | 0.013 | 6.50E-19 | 550.63 |  |
| Rheumatoid arthritis | rs8032939 | C | T | 0.497 | 0.117 | 0.017 | 4.80E-16 | 391.59 |  |
| Rheumatoid arthritis | rs13330176 | A | T | 0.304 | 0.113 | 0.013 | 1.60E-11 | 313.20 |  |
| Rheumatoid arthritis | rs9747973 | T | C | 0.518 | -0.094 | 0.011 | 1.90E-12 | 255.56 |  |
| Rheumatoid arthritis | rs8083786 | G | A | 0.290 | 0.128 | 0.018 | 1.00E-15 | 388.16 |  |
| Rheumatoid arthritis | rs74956615 | A | T | 0.014 | -0.371 | 0.049 | 3.10E-16 | 218.27 |  |
| Rheumatoid arthritis | rs4239702 | C | T | 0.268 | 0.117 | 0.012 | 9.00E-15 | 307.14 |  |
| Rheumatoid arthritis | rs1893592 | C | A | 0.211 | -0.104 | 0.014 | 3.70E-12 | 208.69 |  |
| Rheumatoid arthritis | rs909685 | A | T | 0.494 | 0.113 | 0.018 | 6.30E-14 | 370.17 |  |
| Systemic lupus erythematosus | rs4661543 | G | T | 0.873 | 0.274 | 0.042 | 9.40E-11 | 242.61 |  |
| Systemic lupus erythematosus | rs10912578 | G | A | 0.383 | -0.247 | 0.031 | 1.65E-15 | 423.04 |  |
| Systemic lupus erythematosus | rs12094036 | C | T | 0.082 | -0.329 | 0.058 | 1.37E-08 | 234.26 |  |
| Systemic lupus erythematosus | rs13019891 | T | G | 0.549 | -0.562 | 0.029 | 1.65E-83 | 2646.48 |  |
| Systemic lupus erythematosus | rs2573219 | C | A | 0.087 | 0.588 | 0.043 | 1.13E-42 | 823.86 |  |
| Systemic lupus erythematosus | rs10200680 | T | C | 0.856 | -0.248 | 0.042 | 4.96E-09 | 220.58 |  |
| Systemic lupus erythematosus | rs268124 | T | C | 0.274 | 0.186 | 0.032 | 8.60E-09 | 199.98 |  |
| Systemic lupus erythematosus | rs2459611 | T | C | 0.125 | 0.261 | 0.045 | 7.62E-09 | 216.70 |  |
| Systemic lupus erythematosus | rs34703115 | C | T | 0.033 | -0.616 | 0.105 | 4.08E-09 | 352.13 |  |
| Systemic lupus erythematosus | rs1464446 | T | G | 0.179 | -0.329 | 0.040 | 2.79E-16 | 467.07 |  |
| Systemic lupus erythematosus | rs9852014 | G | A | 0.925 | 0.621 | 0.049 | 2.26E-36 | 801.11 |  |
| Systemic lupus erythematosus | rs13136219 | T | C | 0.620 | -0.174 | 0.028 | 3.50E-10 | 207.24 |  |
| Systemic lupus erythematosus | rs1078324 | A | C | 0.050 | -0.713 | 0.078 | 7.11E-20 | 720.31 |  |
| Systemic lupus erythematosus | rs4388254 | T | C | 0.238 | 0.378 | 0.060 | 3.71E-10 | 781.12 |  |
| Systemic lupus erythematosus | rs6889239 | C | T | 0.462 | 0.278 | 0.032 | 2.19E-18 | 568.43 |  |
| Systemic lupus erythematosus | rs9274357 | T | C | 0.776 | 0.457 | 0.035 | 1.28E-38 | 1117.90 |  |
| Systemic lupus erythematosus | rs7768653 | T | C | 0.402 | -0.207 | 0.030 | 3.11E-12 | 300.00 |  |
| Systemic lupus erythematosus | rs12524498 | T | G | 0.989 | -0.673 | 0.121 | 2.48E-08 | 140.83 |  |
| Systemic lupus erythematosus | rs150180633 | T | C | 0.031 | 0.928 | 0.069 | 2.66E-41 | 767.17 |  |
| Systemic lupus erythematosus | rs7823055 | T | G | 0.424 | -0.351 | 0.029 | 1.64E-34 | 911.19 |  |
| Systemic lupus erythematosus | rs58688157 | G | A | 0.287 | -0.223 | 0.034 | 2.97E-11 | 296.56 |  |
| Systemic lupus erythematosus | rs353608 | G | A | 0.548 | 0.186 | 0.028 | 2.93E-11 | 249.67 |  |
| Systemic lupus erythematosus | rs73050535 | T | C | 0.970 | -0.713 | 0.124 | 9.11E-09 | 432.47 |  |
| Systemic lupus erythematosus | rs1143679 | A | G | 0.085 | 0.582 | 0.040 | 5.03E-48 | 794.56 |  |
| Systemic lupus erythematosus | rs28834423 | C | G | 0.183 | 0.457 | 0.037 | 5.65E-36 | 951.65 |  |
| Systemic lupus erythematosus | rs13332649 | G | A | 0.110 | -0.315 | 0.038 | 5.43E-17 | 282.11 |  |
| Systemic lupus erythematosus | rs143123127 | A | G | 0.050 | 0.470 | 0.084 | 2.23E-08 | 305.31 |  |
| Systemic lupus erythematosus | rs73068668 | A | G | 0.035 | -0.315 | 0.057 | 4.40E-08 | 95.39 |  |
| Colorectal cancer | rs72647484 | C | T | 0.027 | -0.113 | 0.020 | 1.14E-08 | 174.74 |  |
| Colorectal cancer | rs871524 | A | G | 0.202 | -0.078 | 0.012 | 1.46E-10 | 506.21 |  |
| Colorectal cancer | rs12144325 | G | A | 0.869 | 0.096 | 0.016 | 9.53E-10 | 535.43 |  |
| Colorectal cancer | rs10911228 | C | T | 0.326 | -0.085 | 0.011 | 4.36E-14 | 820.76 |  |
| Colorectal cancer | rs6604678 | A | G | 0.416 | 0.084 | 0.011 | 1.76E-13 | 885.60 |  |
| Colorectal cancer | rs79170240 | C | T | 0.083 | -0.118 | 0.020 | 6.86E-09 | 538.78 |  |
| Colorectal cancer | rs6752114 | T | C | 0.723 | 0.078 | 0.012 | 5.81E-11 | 624.32 |  |
| Colorectal cancer | rs13020391 | T | C | 0.296 | -0.082 | 0.012 | 2.23E-12 | 722.08 |  |
| Colorectal cancer | rs35470271 | G | A | 0.147 | 0.090 | 0.015 | 6.17E-09 | 520.16 |  |
| Colorectal cancer | rs9831861 | G | T | 0.433 | 0.067 | 0.011 | 2.38E-09 | 569.80 |  |
| Colorectal cancer | rs1499899 | A | G | 0.494 | -0.066 | 0.011 | 3.85E-09 | 563.16 |  |
| Colorectal cancer | rs60486266 | T | G | 0.833 | -0.085 | 0.015 | 2.98E-08 | 512.95 |  |
| Colorectal cancer | rs4698932 | A | G | 0.330 | -0.063 | 0.011 | 3.66E-08 | 446.13 |  |
| Colorectal cancer | rs2735940 | G | A | 0.473 | 0.079 | 0.011 | 3.34E-12 | 798.37 |  |
| Colorectal cancer | rs72748443 | G | C | 0.746 | 0.107 | 0.012 | 2.88E-18 | 1103.85 |  |
| Colorectal cancer | rs639933 | A | C | 0.315 | -0.064 | 0.012 | 3.08E-08 | 452.50 |  |
| Colorectal cancer | rs2070699 | T | G | 0.356 | 0.068 | 0.011 | 2.79E-09 | 534.38 |  |
| Colorectal cancer | rs112582516 | T | A | 0.675 | 0.127 | 0.019 | 1.68E-11 | 1818.45 |  |
| Colorectal cancer | rs12110366 | T | C | 0.983 | -0.214 | 0.039 | 4.47E-08 | 388.35 |  |
| Colorectal cancer | rs4331968 | T | A | 0.781 | 0.086 | 0.013 | 4.60E-11 | 646.26 |  |
| Colorectal cancer | rs6933790 | C | T | 0.202 | -0.091 | 0.016 | 5.57E-09 | 675.76 |  |
| Colorectal cancer | rs2498529 | A | G | 0.633 | 0.071 | 0.012 | 1.21E-09 | 593.97 |  |
| Colorectal cancer | rs3801081 | G | A | 0.250 | 0.077 | 0.012 | 1.01E-10 | 571.96 |  |
| Colorectal cancer | rs16892766 | C | A | 0.082 | 0.224 | 0.019 | 1.76E-31 | 1949.48 |  |
| Colorectal cancer | rs6983267 | T | G | 0.390 | -0.170 | 0.011 | 2.50E-52 | 3562.01 |  |
| Colorectal cancer | rs1412834 | C | T | 0.310 | -0.077 | 0.011 | 5.35E-12 | 643.46 |  |
| Colorectal cancer | rs12004944 | C | T | 0.015 | -0.295 | 0.054 | 3.71E-08 | 652.65 |  |
| Colorectal cancer | rs11789898 | T | G | 0.115 | 0.086 | 0.015 | 1.56E-08 | 380.71 |  |
| Colorectal cancer | rs7894531 | A | G | 0.256 | -0.111 | 0.012 | 2.52E-20 | 1206.44 |  |
| Colorectal cancer | rs704017 | G | A | 0.451 | 0.102 | 0.012 | 4.87E-18 | 1307.21 |  |
| Colorectal cancer | rs2193352 | G | A | 0.123 | 0.104 | 0.014 | 7.76E-14 | 592.08 |  |
| Colorectal cancer | rs56188434 | A | G | 0.099 | 0.107 | 0.019 | 8.69E-09 | 519.00 |  |
| Colorectal cancer | rs57796856 | T | A | 0.318 | 0.080 | 0.011 | 5.84E-13 | 714.89 |  |
| Colorectal cancer | rs4944940 | A | G | 0.031 | -0.270 | 0.030 | 2.35E-19 | 1136.41 |  |
| Colorectal cancer | rs7130173 | C | A | 0.288 | -0.137 | 0.012 | 1.14E-29 | 1967.77 |  |
| Colorectal cancer | rs10849433 | C | T | 0.473 | 0.092 | 0.013 | 1.89E-12 | 1070.81 |  |
| Colorectal cancer | rs11169578 | A | G | 0.388 | 0.075 | 0.011 | 3.20E-11 | 690.62 |  |
| Colorectal cancer | rs7398375 | G | C | 0.315 | -0.077 | 0.013 | 1.24E-08 | 649.23 |  |
| Colorectal cancer | rs6490019 | G | A | 0.412 | 0.068 | 0.012 | 1.57E-08 | 576.75 |  |
| Colorectal cancer | rs12427846 | C | T | 0.130 | 0.087 | 0.013 | 7.59E-11 | 438.72 |  |
| Colorectal cancer | rs1411715 | G | T | 0.138 | 0.102 | 0.017 | 2.35E-09 | 631.01 |  |
| Colorectal cancer | rs4773183 | A | T | 0.649 | 0.077 | 0.012 | 4.29E-11 | 697.18 |  |
| Colorectal cancer | rs35107139 | C | A | 0.483 | 0.096 | 0.012 | 1.31E-15 | 1166.42 |  |
| Colorectal cancer | rs4901473 | A | G | 0.381 | -0.068 | 0.011 | 3.43E-09 | 551.02 |  |
| Colorectal cancer | rs73376930 | G | A | 0.319 | 0.157 | 0.014 | 9.83E-31 | 2755.42 |  |
| Colorectal cancer | rs144674978 | T | C | 0.988 | 0.358 | 0.051 | 2.25E-12 | 770.16 |  |
| Colorectal cancer | rs4776316 | G | A | 0.221 | -0.071 | 0.013 | 3.23E-08 | 443.99 |  |
| Colorectal cancer | rs7495132 | T | C | 0.147 | 0.103 | 0.017 | 2.11E-09 | 674.20 |  |
| Colorectal cancer | rs35244260 | T | C | 0.461 | -0.099 | 0.015 | 1.03E-11 | 1250.65 |  |
| Colorectal cancer | rs899244 | T | C | 0.213 | 0.079 | 0.014 | 5.81E-09 | 530.35 |  |
| Colorectal cancer | rs1078643 | A | G | 0.450 | 0.091 | 0.014 | 5.53E-11 | 1054.70 |  |
| Colorectal cancer | rs34400960 | G | C | 0.286 | 0.074 | 0.013 | 1.49E-08 | 575.39 |  |
| Colorectal cancer | rs7226855 | G | A | 0.395 | -0.187 | 0.011 | 4.66E-62 | 4326.50 |  |
| Colorectal cancer | rs10409772 | A | C | 0.328 | 0.121 | 0.021 | 1.70E-08 | 1648.18 |  |
| Colorectal cancer | rs73039426 | T | C | 0.935 | -0.224 | 0.028 | 6.38E-16 | 1548.15 |  |
| Colorectal cancer | rs4803457 | C | T | 0.453 | 0.070 | 0.012 | 1.74E-09 | 624.72 |  |
| Colorectal cancer | rs961253 | A | C | 0.292 | 0.101 | 0.012 | 1.91E-18 | 1082.89 |  |
| Colorectal cancer | rs6085662 | C | G | 0.292 | 0.078 | 0.012 | 1.69E-11 | 634.77 |  |
| Colorectal cancer | rs6066825 | G | A | 0.485 | -0.093 | 0.012 | 4.49E-15 | 1095.03 |  |
| Colorectal cancer | rs370862 | T | A | 0.598 | 0.079 | 0.011 | 2.23E-12 | 776.31 |  |
| Colorectal cancer | rs1741640 | C | T | 0.334 | 0.145 | 0.014 | 9.80E-26 | 2409.13 |  |
| Abbreviations: EAF, effect allele frequency; SE, standard error; SNP, single-nucleotide polymorphism. # The F-statistic was estimated using the following formula: R^2(N-2)/(1-R^2), where N is the sample size. | | | | | | | | |  |
|  |  |  |  |  |  |  |  |  |  |
