# Supplementary material for: Bidirectional Mendelian Randomization Analysis Reveals Causal Associations Between Autoimmune Diseases and Colorectal Cancer
Source: World J Oncol. 2026 Mar 5;17(2):256–67. doi: 10.14740/wjon2732 (PMC12978415; doi:10.14740/wjon2732)
Supplement: Suppl 2 — MR analyses of a causal association between genetic liability to autoimmune diseases and colorectal cancer. [file wjon-17-02-256-s002.docx]

| **Suppl 2. MR analyses of a causal association between genetic liability to autoimmune diseases and colorectal cancer.** | | | | | | | | | | | |
| --- | --- | --- | --- | --- | --- | --- | --- | --- | --- | --- | --- |
| **Outcome** | **Exposure** | **SNPs（n）** | **IVW** | | | **MR-Egger** | | | **Weighted median** | | |
|  |  |  | **OR (95% CI)** | **SE** | ***P*** | **OR (95% CI)** | **SE** | ***P*** | **OR (95% CI)** | **SE** | ***P*** |
| Colorectal cancer | Rheumatoid arthritis | 59 | 1.061 (1.020, 1.111) | 0.022 | **0.004** | 1.047 (1.011, 1.111) | 0.034 | 0.201 | 1.035 (1.002, 1.086) | 0.025 | 0.167 |
| Colorectal cancer | Systemic lupus erythematosus | 28 | 0.996 (0.978, 1.016) | 0.010 | 0.714 | 1.016 (0.971, 1.062) | 0.023 | 0.504 | 0.988 (0.962, 1.014) | 0.013 | 0.361 |
| Colorectal cancer | Celiac disease | 11 | 0.938 (0.886, 0.993) 0.8861562， 0.9931199） | 0.029 | **0.028** | 0.958 (0.780, 1.178) | 0.105 | 0.697 | 0.954 (0.900, 1.011) | 0.030 | 0.109 |
| Colorectal cancer | Asthma | 18 | 1.068 (0.966, 1.181) | 0.051 | 0.201 | 1.211 (0.849, 1.726) | 0.181 | 0.306 | 1.054 (0.958, 1.160) | 0.049 | 0.278 |
| Colorectal cancer | Multiple sclerosis | 89 | 1.001 (0.977, 1.025) | 0.012 | 0.945 | 1.003 (0.967, 1.040) | 0.019 | 0.881 | 0.987 (0.955, 1.019) | 0.017 | 0.419 |
| Colorectal cancer | Gout | 6 | 1.000 (0.961, 1.041) | 0.020 | 0.987 | 1.011 (0.936, 1.092) | 0.039 | 0.796 | 0.996 (0.951, 1.043) | 0.024 | 0.866 |
| Colorectal cancer | Ankylosing spondylitis | 11 | 0.995 (0.971, 1.018) | 0.012 | 0.656 | 0.993 (0.946, 1.041) | 0.024 | 0.771 | 0.993 (0.964, 1.022) | 0.015 | 0.634 |
| Colorectal cancer | Eczema | 11 | 1.007 (0.945, 1.074) | 0.033 | 0.824 | 1.015 (0.768, 1.341) | 0.142 | 0.921 | 1.008 (0.929, 1.093) | 0.041 | 0.850 |
| Abbreviations: OR, Odds ratio; CI, Confidence internal; SE, Standard error; IVW, Inverse-variance weighted. Bold indicates statistically significant difference (*P* < 0.05). | | | | | | | | | | | |
