# Supplementary material for: Bidirectional Mendelian Randomization Analysis Reveals Causal Associations Between Autoimmune Diseases and Colorectal Cancer
Source: World J Oncol. 2026 Mar 5;17(2):256–67. doi: 10.14740/wjon2732 (PMC12978415; doi:10.14740/wjon2732)
Supplement: Suppl 3 — Scatter plots of the association between autoimmune diseases and colorectal cancer. [file wjon-17-02-256-s003.pptx]

## Slide 1
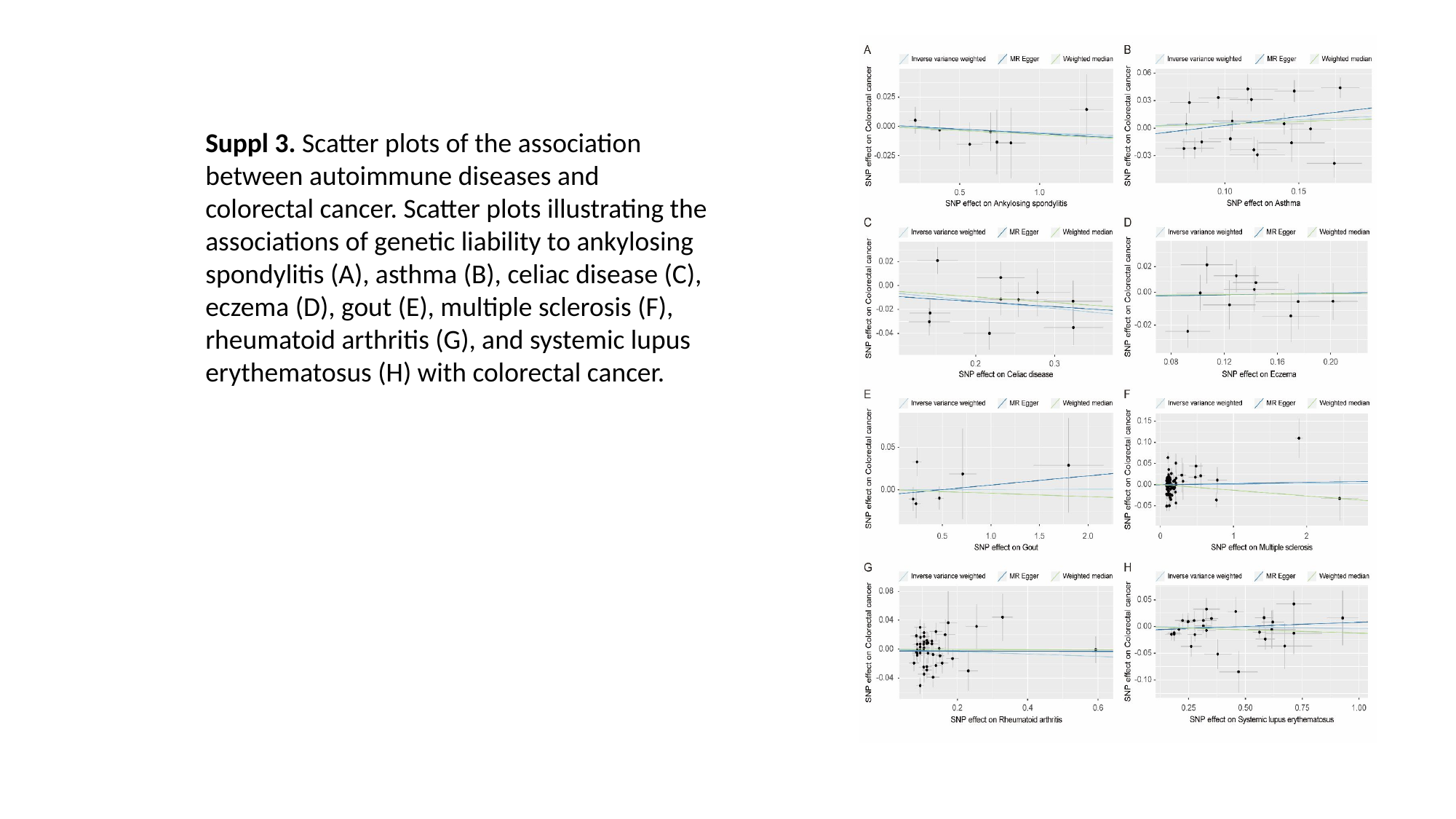

Suppl 3. Scatter plots of the association between autoimmune diseases and colorectal cancer. Scatter plots illustrating the associations of genetic liability to ankylosing spondylitis (A), asthma (B), celiac disease (C), eczema (D), gout (E), multiple sclerosis (F), rheumatoid arthritis (G), and systemic lupus erythematosus (H) with colorectal cancer.
