# Supplementary material for: Bidirectional Mendelian Randomization Analysis Reveals Causal Associations Between Autoimmune Diseases and Colorectal Cancer
Source: World J Oncol. 2026 Mar 5;17(2):256–67. doi: 10.14740/wjon2732 (PMC12978415; doi:10.14740/wjon2732)
Supplement: Suppl 4 — Sensitivity analyses of a causal association between genetic liability to autoimmune diseases and colorectal cancer. [file wjon-17-02-256-s004.docx]

**Suppl 4.** Sensitivity analyses of a causal association between genetic liability to autoimmune diseases and colorectal cancer

| **Outcome** | **Exposure** | **Cochran’s Q statistic (heterogeneity)** | | **MR-Egger intercept test (pleiotropy, *P*)** | **CAUSE (*P*)** |
| --- | --- | --- | --- | --- | --- |
|  |  | **IVW ( *P* )** | **MR Egger ( *P* )** |  |  |
| Colorectal cancer | Rheumatoid arthritis | **0.000** | **0.000** | 0.720 | **0.002** |
| Colorectal cancer | Systemic lupus erythematosus | 0.190 | 0.188 | 0.367 | 0.641 |
| Colorectal cancer | Celiac disease | **0.016** | **0.010** | 0.838 | **0.021** |
| Colorectal cancer | Asthma | **0.000** | **0.000** | 0.479 | 0.054 |
| Colorectal cancer | Multiple sclerosis | **0.000** | **0.000** | 0.889 | 0.211 |
| Colorectal cancer | Gout | 0.274 | 0.186 | 0.759 | 0.312 |
| Colorectal cancer | Ankylosing spondylitis | 0.960 | 0.915 | 0.925 | **0.032** |
| Colorectal cancer | Eczema | 0.314 | 0.234 | 0.959 | **0.026** |
| Abbreviations: IVW, Inverse-variance weighted. Bold indicates statistically significant difference (*P* < 0.05). | | | | |  |
