# Supplementary material for: Bidirectional Mendelian Randomization Analysis Reveals Causal Associations Between Autoimmune Diseases and Colorectal Cancer
Source: World J Oncol. 2026 Mar 5;17(2):256–67. doi: 10.14740/wjon2732 (PMC12978415; doi:10.14740/wjon2732)
Supplement: Suppl 5 — Funnel plot analysis for the association between autoimmune diseases and colorectal cancer. [file wjon-17-02-256-s005.pptx]

## Slide 1
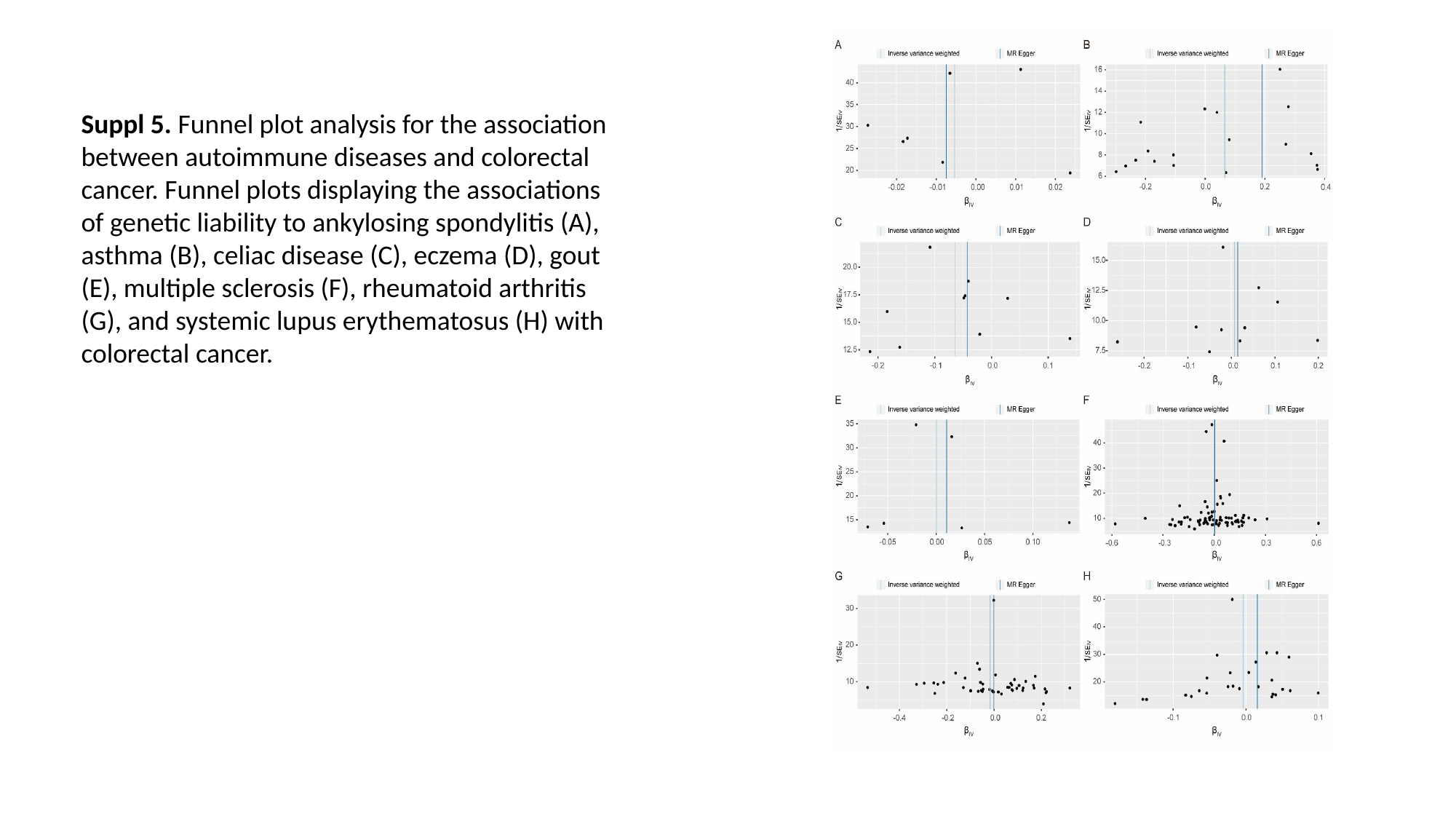

Suppl 5. Funnel plot analysis for the association between autoimmune diseases and colorectal cancer. Funnel plots displaying the associations of genetic liability to ankylosing spondylitis (A), asthma (B), celiac disease (C), eczema (D), gout (E), multiple sclerosis (F), rheumatoid arthritis (G), and systemic lupus erythematosus (H) with colorectal cancer.
