# Supplementary material for: Bidirectional Mendelian Randomization Analysis Reveals Causal Associations Between Autoimmune Diseases and Colorectal Cancer
Source: World J Oncol. 2026 Mar 5;17(2):256–67. doi: 10.14740/wjon2732 (PMC12978415; doi:10.14740/wjon2732)
Supplement: Suppl 6 — Forest plots of leave-one-out analyses of the association between autoimmune diseases and colorectal cancer. [file wjon-17-02-256-s006.pptx]

## Slide 1
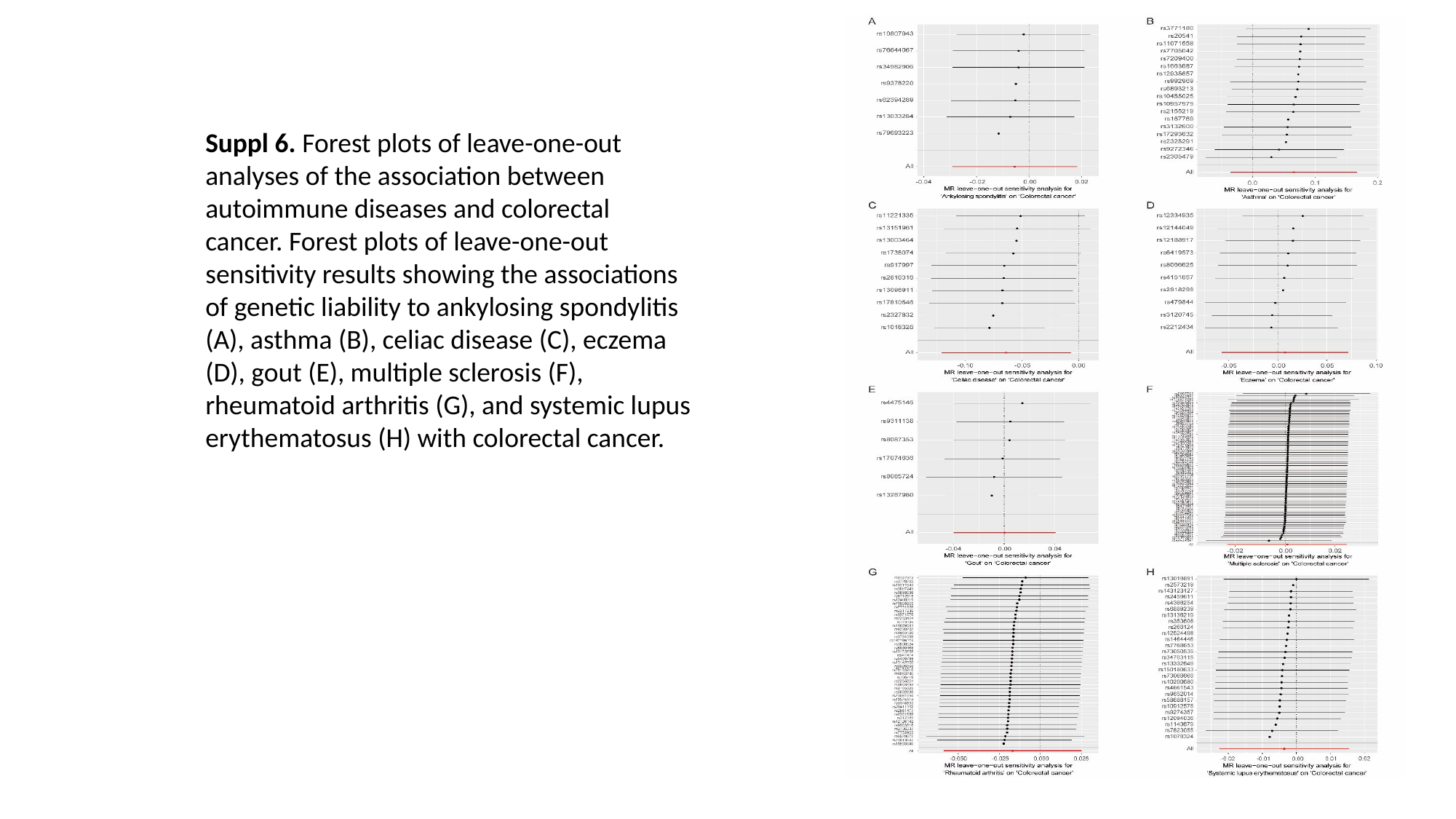

Suppl 6. Forest plots of leave-one-out analyses of the association between autoimmune diseases and colorectal cancer. Forest plots of leave-one-out sensitivity results showing the associations of genetic liability to ankylosing spondylitis (A), asthma (B), celiac disease (C), eczema (D), gout (E), multiple sclerosis (F), rheumatoid arthritis (G), and systemic lupus erythematosus (H) with colorectal cancer.
