# Supplementary material for: Bidirectional Mendelian Randomization Analysis Reveals Causal Associations Between Autoimmune Diseases and Colorectal Cancer
Source: World J Oncol. 2026 Mar 5;17(2):256–67. doi: 10.14740/wjon2732 (PMC12978415; doi:10.14740/wjon2732)
Supplement: Suppl 7 — MR analyses of a causal association between genetic liability to colorectal cancer and autoimmune diseases. [file wjon-17-02-256-s007.docx]

**Suppl 7.** MR analyses of a causal association between genetic liability to colorectal cancer and autoimmune diseases.

| **Outcome** | **Exposure** | **SNPs（n）** | **IVW** | | | **MR-Egger** | | | **Weighted median** | | |
| --- | --- | --- | --- | --- | --- | --- | --- | --- | --- | --- | --- |
|  |  |  | **OR (95% CI)** | **SE** | ***P*** | **OR (95% CI)** | **SE** | ***P*** | **OR (95% CI)** | **SE** | ***P*** |
| Rheumatoid arthritis | Colorectal cancer | 61 | 1.000 (0.949, 1.055) | 0.027 | 0.989 | 0.934 (0.809, 1.079) | 0.074 | 0.361 | 0.955 (0.884, 1.032) | 0.040 | 0.249 |
| Systemic lupus erythematosus | Colorectal cancer | 61 | 1.115 (0.998, 1.244) | 0.056 | 0.053 | 1.095 (0.795, 1.508) | 0.163 | 0.582 | 1.190 (1.032, 1.372) | 0.073 | **0.016** |
| Celiac disease | Colorectal cancer | 61 | NA | NA | NA | NA | NA | NA | NA | NA | NA |
| Asthma | Colorectal cancer | 61 | NA | NA | NA | NA | NA | NA | NA | NA | NA |
| Multiple sclerosis | Colorectal cancer | 61 | 0.964 (0.900, 1.032) | 0.035 | 0.291 | 1.049 (0.867, 1.268) | 0.097 | 0.625 | 0.962 (0.881, 1.050) | 0.045 | 0.383 |
| Gout | Colorectal cancer | 61 | NA | NA | NA | NA | NA | NA | NA | NA | NA |
| Ankylosing spondylitis | Colorectal cancer | 61 | 1.015 (0.905, 1.138) | 0.058 | 0.803 | 0.866 ( 0.640, 1.170) | 0.154 | 0.352 | 0.959 (0.810, 1.136) | 0.086 | 0.629 |
| Eczema | Colorectal cancer | 61 | 1.002 (0.936, 1.073) | 0.035 | 0.948 | 0.928 (0.763, 1.128) | 0.100 | 0.455 | 1.010 ( 0.928, 1.099) | 0.043 | 0.820 |
| Abbreviations: OR, Odds ratio; CI, Confidence internal; SE, Standard error; IVW, Inverse-variance weighted. Bold indicates statistically significant difference (*P* < 0.05). NA indicates that there is an insufficient number of single-nucleotide polymorphisms to compute MR estimates for specific sensitivity analyses. | | | | | | | | | | | |
