# Supplementary material for: Bidirectional Mendelian Randomization Analysis Reveals Causal Associations Between Autoimmune Diseases and Colorectal Cancer
Source: World J Oncol. 2026 Mar 5;17(2):256–67. doi: 10.14740/wjon2732 (PMC12978415; doi:10.14740/wjon2732)
Supplement: Suppl 8 — Scatter plots of the association between colorectal cancer and autoimmune diseases. [file wjon-17-02-256-s008.pptx]

## Slide 1
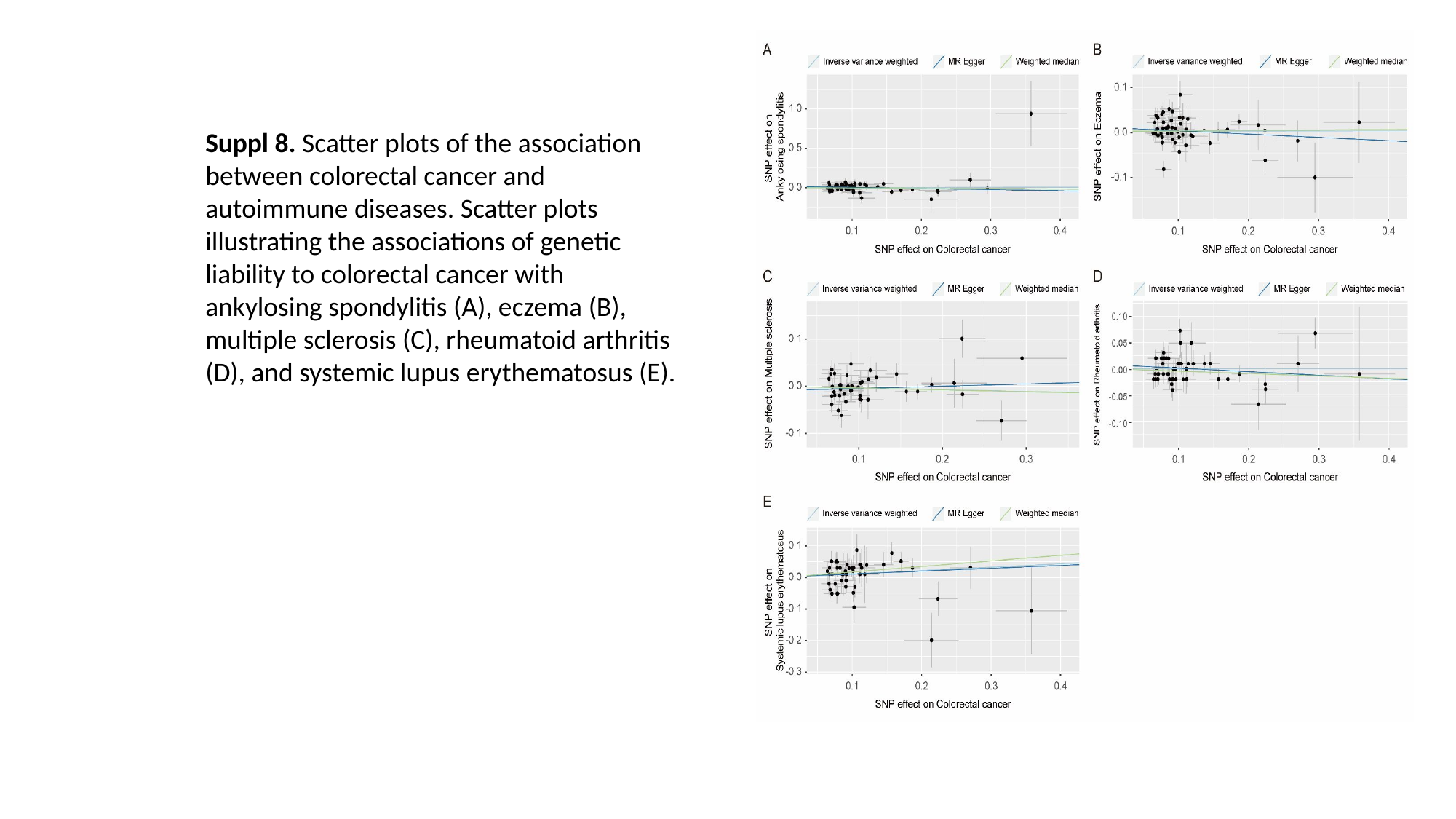

Suppl 8. Scatter plots of the association between colorectal cancer and autoimmune diseases. Scatter plots illustrating the associations of genetic liability to colorectal cancer with ankylosing spondylitis (A), eczema (B), multiple sclerosis (C), rheumatoid arthritis (D), and systemic lupus erythematosus (E).
