# Supplementary material for: Bidirectional Mendelian Randomization Analysis Reveals Causal Associations Between Autoimmune Diseases and Colorectal Cancer
Source: World J Oncol. 2026 Mar 5;17(2):256–67. doi: 10.14740/wjon2732 (PMC12978415; doi:10.14740/wjon2732)
Supplement: Suppl 10 — Forest plots of leave-one-out analyses of the association between colorectal cancer and autoimmune diseases. [file wjon-17-02-256-s010.pptx]

## Slide 1
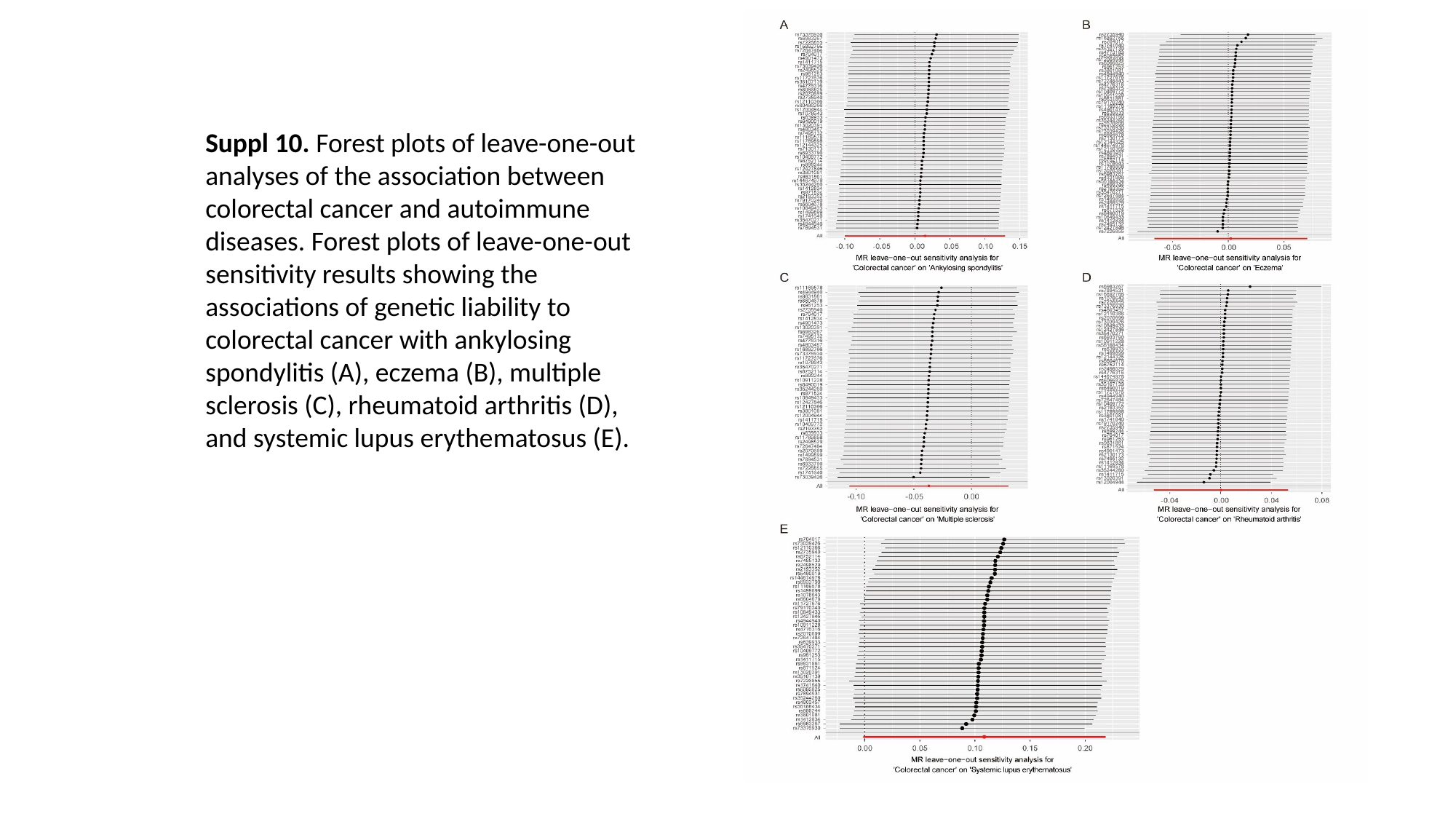

Suppl 10. Forest plots of leave-one-out analyses of the association between colorectal cancer and autoimmune diseases. Forest plots of leave-one-out sensitivity results showing the associations of genetic liability to colorectal cancer with ankylosing spondylitis (A), eczema (B), multiple sclerosis (C), rheumatoid arthritis (D), and systemic lupus erythematosus (E).
