# Supplementary material for: Bidirectional Mendelian Randomization Analysis Reveals Causal Associations Between Autoimmune Diseases and Colorectal Cancer
Source: World J Oncol. 2026 Mar 5;17(2):256–67. doi: 10.14740/wjon2732 (PMC12978415; doi:10.14740/wjon2732)
Supplement: Suppl 11 — Sensitivity analyses of a causal association between genetic liability to colorectal cancer and autoimmune diseases. [file wjon-17-02-256-s011.docx]

**Suppl 11.** Sensitivity analyses of a causal association between genetic liability to colorectal cancer and autoimmune diseases.

| **Outcome** | **Exposure** | **Cochran’s Q statistic (heterogeneity)** | | **MR-Egger intercept test (pleiotropy, *P*)** |  |
| --- | --- | --- | --- | --- | --- |
|  |  | **IVW ( *P* )** | **MR Egger ( *P* )** |  |  |
| Rheumatoid arthritis | Colorectal cancer | 0.067 | 0.069 | 0.324 |  |
| Systemic lupus erythematosus | Colorectal cancer | 0.078 | 0.064 | 0.908 |  |
| Celiac disease | Colorectal cancer | NA | NA | NA |  |
| Asthma | Colorectal cancer | NA | NA | NA |  |
| Multiple sclerosis | Colorectal cancer | **0.037** | **0.037** | 0.354 |  |
| Gout | Colorectal cancer | NA | NA | NA |  |
| Ankylosing spondylitis | Colorectal cancer | 0.763 | 0.774 | 0.269 |  |
| Eczema | Colorectal cancer | **0.006** | **0.006** | 0.411 |  |
| Abbreviations: IVW, Inverse-variance weighted. Bold indicates statistically significant difference (*P* < 0.05). NA indicates that there is an insufficient number of single-nucleotide polymorphisms to compute MR estimates for specific sensitivity analyses. | | | | |  |
|  |  |  |  |  |  |
